# Age‐related decline in cortical inhibitory tone strengthens motor memory

**DOI:** 10.1016/j.neuroimage.2021.118681

**Published:** 2021-12-15

**Authors:** Pierre Petitet, Gershon Spitz, Uzay E. Emir, Heidi Johansen-Berg, Jacinta O’Shea

**Affiliations:** a Wellcome Centre for Integrative Neuroimaging, FMRIB Centre, Nuffield Department of Clinical Neurosciences (NDCN), John Radcliffe Hospital, Headington, Oxford, United Kingdom; b Centre de Recherche en Neurosciences de Lyon, Equipe Trajectoires, Inserm UMR-S 1028, CNRS UMR 5292, Université Lyon 1, Bron, France; c Turner Institute for Brain and Mental Health, Monash University, Melbourne, Australia; d School of Health Sciences, Purdue University, West Lafayette, Indiana, USA; e Weldon School of Biomedical Engineering, Purdue University, West Lafayette, Indiana, USA; f Wellcome Centre for Integrative Neuroimaging, Oxford Centre for Human Brain Activity (OHBA), University of Oxford Department of Psychiatry, Warneford Hospital, Warneford Lane, Oxford, United Kingdom

**Keywords:** Sensorimotor adaptation, Ageing, Excitation:inhibition ratio

## Abstract

Ageing disrupts the finely tuned excitation/inhibition balance (E:I) across cortex via a natural decline in inhibitory tone (γ-amino butyric acid, GABA), causing functional decrements. However, in young adults, experimentally lowering GABA in sensorimotor cortex enhances a specific domain of sensorimotor function: adaptation memory. Here, we tested the hypothesis that as sensorimotor cortical GABA declines naturally with age, adaptation memory would increase, and the former would explain the latter. Results confirmed this prediction. To probe causality, we used brain stimulation to further lower sensorimotor cortical GABA during adaptation. Across individuals, how stimulation changed memory depended on sensorimotor cortical E:I. In those with low E:I, stimulation increased memory; in those with high E:I stimulation reduced memory. Thus, we identified a form of motor memory that is naturally strengthened by age, depends causally on sensorimotor cortex neurochemistry, and may be a potent target for motor skill preservation strategies in healthy ageing and neurorehabilitation.

## Highlights

• Older age is associated with lower GABAergic inhibition in primary motor cortex (M1).
• Lower GABAergic inhibition in M1 is associated with more persistent adaptation memory.
• This explains why older age is associated with more persistent adaptation memory.
• The way neurostimulation changes adaptation memory depends on M1 neurochemistry.

## Graphical abstract

**Figure:**
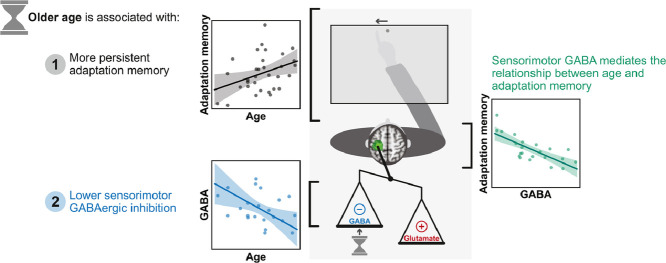

## 1 Introduction

Motor capacities decline with age (Hunter, Pereira, Keenan, 2016, Krampe, 2002). As the brain and body become older, movements lose speed (Bedard, Nichols, Barbosa, Schachar, Logan, Tannock, 2002, Jiménez-Jiménez, Calleja, Alonso-Navarro, Rubio, Navacerrada, de-la Fuente, Plaza-Nieto, Arroyo-Solera, García-Ruiz, García-Martín, Agúndez, 2011), strength (Frontera et al., 2000), and coordination (Serrien et al., 2000). This natural loss of function is exacerbated by motor disorders which rise sharply with age (e.g. stroke, sarcopenia, Parkinsonism). As the elderly population increases (Leeson, 2018), there is a need for strategies to counteract and compensate for age-related motor decline.

During ageing, the motor system must adapt continuously to ongoing neuro-musculo-skeletal change. Brain plasticity enables this. Plasticity is essential to learn new motor skills, adapt and retain existing ones, and to rehabilitate functions impaired by disease (Dayan, Cohen, 2011, Sampaio-Baptista, Sanders, Johansen-Berg, 2018). Thus plasticity plays an important role in mitigating age-related motor decline (McNeil, Rice, 2018, Rozycka, Liguz-Lecznar, 2017).

Unfortunately, plasticity also declines with age (Burke and Barnes, 2006), especially in the motor domain (Bhandari, Radhu, Farzan, Mulsant, Rajji, Daskalakis, Blumberger, 2016, Freitas, Farzan, Pascual-Leone, 2013, Rogasch, Dartnall, Cirillo, Nordstrom, Semmler, 2009). A major cause is the dysregulation of the finely tuned balance between cortical excitation and inhibition (E:I) (Rozycka and Liguz-Lecznar, 2017). Across cortex, E:I is disrupted because γ-aminobutyric acid (GABA) – the major inhibitory neurotransmitter – has predominantly been reported to decline with age, both in animals (David-Jürgens, Dinse, 2009, Schmidt, Redecker, Bruehl, Witte, 2010) and humans (Bhandari, Radhu, Farzan, Mulsant, Rajji, Daskalakis, Blumberger, 2016, Cheng, Lin, 2013, Gao, Edden, Li, Puts, Wang, Liu, Zhao, Wang, Bai, Zhao, Wang, Barker, 2013, Heise, Zimerman, Hoppe, Gerloff, Wegscheider, Hummel, 2013, Hermans, Leunissen, Pauwels, Cuypers, Peeters, Puts, Edden, Swinnen, 2018, King, Rumpf, Verbaanderd, Heise, Dolfen, Sunaert, Doyon, Classen, Mantini, Puts, Edden, Albouy, Swinnen, 2020, Lenz, Tegenthoff, Kohlhaas, Stude, Höffken, Gatica Tossi, Kalisch, Dinse, 2012, Levin, Fujiyama, Boisgontier, Swinnen, Summers, 2014, Mooney, Cirillo, Byblow, 2017, Oliviero, Profice, Tonali, Pilato, Saturno, Dileone, Ranieri, Di Lazzaro, 2006, Peinemann, Lehner, Conrad, Siebner, 2001, Porges, Woods, Edden, Puts, Harris, Chen, Garcia, Seider, Lamb, Williamson, Cohen, 2017; although see: Cuypers, Hehl, van Aalst, Chalavi, Mikkelsen, Van Laere, Dupont, Mantini, Swinnen, 2021, Hermans, Levin, Maes, van Ruitenbeek, Heise, Edden, Puts, Peeters, King, Meesen, Leunissen, Swinnen, Cuypers, 2018). Regional decline of cortical GABA causes a loss of inhibitory tone, and this is associated with decrements in functions localized to the affected regions (Chamberlain, Gagnon, Lalwani, Cassady, Simmonite, Seidler, Taylor, Weissman, Park, Polk, 2021, Marenco, Meyer, van der Veen, Zhang, Kelly, Shen, Weinberger, Dickinson, Berman, 2018, Simmonite, Carp, Foerster, Ossher, Petrou, Weissman, Polk, 2019). For example, in somatosensory cortex lower GABA (i.e. higher E:I) is associated with poorer tactile discrimination, both in young and old adults (Kolasinski, Logan, Hinson, Manners, Divanbeighi Zand, Makin, Emir, Stagg, 2017, Lenz, Tegenthoff, Kohlhaas, Stude, Höffken, Gatica Tossi, Kalisch, Dinse, 2012). In primary motor cortex (M1), age-related decline of inhibitory tone is associated with poorer upper-limb dexterity (Heise et al., 2013), postural imbalance (Papegaaij, Taube, Hogenhout, Baudry, Hortobágyi, 2014, Swanson, Fling, 2018), impaired ability to suppress automatic responses (Hermans et al., 2018a), and slower motor sequence learning (King et al., 2020).

By contrast, here we tested the hypothesis that, as M1 GABA declines with age, a specific form of upper limb motor function – adaptation memory – would *increase*. Across the lifespan, adaptation is that property of the sensorimotor system that enables individuals to counteract perturbations by adjusting their movements and thus maintain successful motor performance (Franklin, Wolpert, 2011, Wolpert, Diedrichsen, Flanagan, 2011). After this form of learning has taken place and the perturbation is removed, adaptation memory is expressed as an *after-effect* (AE) – a movement bias in the direction opposite the perturbation. The strength of adaptation memory is indexed by the persistence over time of this AE. There is a wealth of evidence that while older adults often demonstrate deficits during exposure to a sensorimotor perturbation (i.e. slower error reduction; Anguera, Reuter-Lorenz, Willingham, Seidler, 2011, Bock, 2005, Buch, Young, Contreras-Vidal, 2003, Fernández-Ruiz, Hall, Vergara, Díaz, 2000, Huang, Ahmed, 2014, Panouillères, Joundi, Brittain, Jenkinson, 2015, Vandevoorde, Orban de Xivry, 2019), following removal of the perturbation the AE is preserved (Bock, 2005, Buch, Young, Contreras-Vidal, 2003, Hegele, Heuer, 2008, Panouillères, Joundi, Brittain, Jenkinson, 2015, Roller, Cohen, Kimball, Bloomberg, 2002, Vandevoorde, Orban de Xivry, 2019) or even increased (Fernández-Ruiz, Hall, Vergara, Díaz, 2000, Nemanich, Earhart, 2015, Wolpe, Ingram, Tsvetanov, Henson, Wolpert, Tyler, Brayne, Bullmore, Calder, Cusack, Dalgleish, Duncan, Matthews, Marslen-Wilson, Shafto, Campbell, Cheung, Davis, Geerligs, Kievit, McCarrey, Mustafa, Price, Samu, Taylor, Treder, van Belle, Williams, Bates, Emery, Erzinçlioglu, Gadie, Gerbase, Georgieva, Hanley, Parkin, Troy, Auer, Correia, Gao, Green, Henriques, Allen, Amery, Amunts, Barcroft, Castle, Dias, Dowrick, Fair, Fisher, Goulding, Grewal, Hale, Hilton, Johnson, Johnston, Kavanagh-Williamson, Kwasniewska, McMinn, Norman, Penrose, Roby, Rowland, Sargeant, Squire, Stevens, Stoddart, Stone, Thompson, Yazlik, Barnes, Dixon, Hillman, Mitchell, Villis, Rowe, 2020) compared to younger adults (although see: Malone and Bastian, 2016). From a neurochemical perspective, previous work showed that experimentally lowering M1 inhibitory tone during adaptation via brain stimulation had no influence on the rate of adaptation but increased persistence of the AE in young adults (Galea, Vazquez, Pasricha, Orban de Xivry, Celnik, 2010, O’Shea, Revol, Cousijn, Near, Petitet, Jacquin-Courtois, Johansen-Berg, Rode, Rossetti, 2017). Here, we reasoned that if AE retention depends *causally* on M1 inhibitory tone, then this form of memory may increase naturally with age owing to an age-related M1 GABA decline.

This hypothesis was confirmed in a cross-sectional study of thirty-two healthy older adults (mean age: 67.46 years, *s.d.*: 8.07). Using magnetic resonance spectroscopy (MRS) to quantify neurochemistry, we showed that M1 GABA declines with age. Using prism adaptation (PA; von Helmholtz, 1867), we showed that retention increases with age. A mediation analysis subsequently confirmed that as GABA declines with age, adaptation memory increases, and the former explains the latter. To demonstrate causality, we intervened experimentally with excitatory anodal transcranial direct current stimulation (a-tDCS) – to try and further lower M1 GABA (Antonenko, Schubert, Bohm, Ittermann, Aydin, Hayek, Grittner, Flöel, 2017, Kim, Stephenson, Morris, Jackson, 2014, Stagg, Best, Stephenson, O’Shea, Wylezinska, Kincses, Morris, Matthews, Johansen-Berg, 2009) and thus further increase adaptation memory. On average, stimulation did not increase memory in this age group. Rather, a moderation analysis showed that how stimulation changed memory depended on individuals’ motor cortical E:I. Stimulation increased retention in individuals with low E:I, but decreased retention in individuals with high E:I.

In summary, we identified a specific domain of motor functional plasticity that improves with age, as a natural consequence of motor cortical inhibitory decline. This memory function can be further enhanced by neurostimulation, but only in individuals least affected by age-related dysregulation of motor cortical E:I. These findings challenge the prevailing view of ageing as inevitable functional decline. Whereas learning of new motor skills may decline, the capacity to maintain adaptation of existing skills improves naturally with age. That adaptation memory is enhanced naturally with age indicates it may have untapped potential as a target for training strategies that aim to preserve, improve or restore motor function in healthy or pathological ageing (e.g. prism therapy for visuospatial neglect rehabilitation; O’Shea, Revol, Cousijn, Near, Petitet, Jacquin-Courtois, Johansen-Berg, Rode, Rossetti, 2017, Rossetti, Rode, Pisella, Farné, Li, Boisson, Perenin, 1998).

## 2 Materials and methods

### 2.1 Participants

Thirty two right handed men aged between 49 and 81 (mean age: 67.5 years, *s.d.*: 8.1) participated in this study. All were screened to rule out any personal or family history of neurological or psychiatric disorder and safety contraindications for the MRS and tDCS measurements. The screening was performed by one of the experimenters, and participants’ medical history was determined by self-report. Written informed consent was provided by all participants. The study was approved by the U.K. NHS Research Ethics Committee (Oxford A; REC reference number: 13/SC/0163). In Experiment 1, all participants (n=32) performed prism adaptation (PA) and tests of short (10-minutes) and long-term (24-hours) retention. A sub-sample underwent a MRS scan to measure neurochemistry in left sensorimotor cortex (n=22) and in an anatomical control volume in occipital cortex (n=20; Fig. S2). A sub-sample consented to also participate in Experiment 2 (n=25), consisting of two weekly sessions of PA combined with anodal/sham tDCS to M1. Full details of which measurements were obtained for each individual are in Table S1.

In Experiment 1, the sample size (n=32) was determined based on a power analysis run in G*Power (Faul et al., 2007) (Version 3.1.9.2), informed by previous investigations of the association between behaviour and age-related GABA change within the motor domain (Heise, Zimerman, Hoppe, Gerloff, Wegscheider, Hummel, 2013, Hermans, Leunissen, Pauwels, Cuypers, Peeters, Puts, Edden, Swinnen, 2018). The average effect size across these studies was |ρ|=0.52. To detect an effect of this size requires a minimum sample of n=19 with probability of a Type I error α=0.05, and power (1−β)=0.80 (based on a priori one-tailed correlational analysis). Our sample sizes (n=32 for behavioural analyses; n=20 for neurochemistry analyses) therefore had adequate power. In Experiment 2, sample size was determined based on a comparable power analysis informed by the stimulation effect size reported in our previous work (O’Shea et al., 2017). In that study, left M1 a-tDCS enhanced long-term retention up to four days after adaptation, with an effect size of d=0.73. The minimum sample size required to detect an effect of d=0.73 with probability of a Type I error α=0.05, and power (1−β)=0.80 was n=14 (based on a one-tailed difference of two dependent means). To allow for potential dropouts, twenty-six participants were recruited. One participant was lost to retention follow-up and was therefore not included in the final sample of n=25.

### 2.2 Prism adaptation protocol

In both experiments, PA was performed using a purpose-built automated apparatus (Fig. S1a). Participants sat with their head fixed in a chinrest, viewing a 32-inch horizontal touchscreen through a Liquid Crystal Display (LCD) shutter (Dispersion film, Liquid Crystal Technologies, Ohio, USA). The touchscreen was used to present the visual targets and record reach endpoints, and the LCD shutter was used to control visual feedback of the screen and limb. A button was attached to the pole of the chinrest and served as a starting position for all pointing movements. Participants were instructed to keep the button pressed at all times, and to only release it when initiating a reaching movement towards a target. On after-effect (AE) trials only, the release of the button triggered the LCD shutter to turn opaque, thus occluding visual feedback of endpoint accuracy. In addition, a fixed shutter prevented participants from seeing their limb at the starting position and during the first third of their reaching trajectory. Participants were instructed to not slide their finger across the surface of the touchscreen but to instead touch the screen only at the end of their reaching movement. Pointing errors were calculated as the angle formed between a straight line joining the starting position and the target, and a straight line joining the starting position and the recorded landing position. By convention, errors in the direction of the prismatic shift (rightward/clockwise) were coded as positive, while errors in the opposite direction (leftward/counterclockwise) were coded as negative. The task was programmed in MATLAB version 2014b (MathWorks; https://uk.mathworks.com) using Psychtoolbox (Kleiner et al., 2007) version 3, run on a MacBook Pro laptop. On each trial an audio voice recording instructed participants to reach and point with their right index finger at the target presented on the touchscreen. The target could either be located at the centre of the screen (open-loop trials) or 10 cm to the left or right (closed-loop trials). The distance between participants’ eyes and the central target was  57 cm.

During PA participants alternated between two types of task block: closed-loop pointing (CLP) and open-loop pointing (OLP). On closed-loop trials, participants wore 10${}^{\circ }$ right-shifting prism goggles (glacier goggles: Julbo, Longchaumois, France; lenses: OptiquePeter, Lyon, France) and were instructed to make rapid reaching movements (mean movement duration: 452 ms, *s.d.*: 119 ms) to either the left or right target in a pseudo-randomised order. Participants were trained to keep their finger at the landing position and correct their movement on the next trial as needed. To limit strategic adjustments and “in-flight” error correction (Redding, Wallace, 1996, Redding, Wallace, 2001) visual feedback of the first third of each reaching movement was occluded with the fixed shutter, as in previous work (Inoue, Uchimura, Karibe, O’Shea, Rossetti, Kitazawa, 2015, O’Shea, Revol, Cousijn, Near, Petitet, Jacquin-Courtois, Johansen-Berg, Rode, Rossetti, 2017, O’Shea, Gaveau, Kandel, Koga, Susami, Prablanc, Rossetti, 2014). At the end of every trial, visual feedback of the landing position lasted for 500 ms after the touch was recorded. After this time, the LCD shutter turned opaque and participants had to return to the starting position (i.e. press and hold the button) without visual feedback of their hand. This procedure limited prism exposure to the reaching movement as opposed to the return movement. On open-loop trials, prisms were removed and participants were instructed to point at the central target. Accuracy was emphasized over speed (mean movement duration: 799 ms, s.d.: 135 ms). Visual feedback was prevented on each trial by the LCD shutter turning opaque at reach onset, thus occluding vision of the target, reach and endpoint error, and return movement. This enabled the leftward AE to be measured without participants actively de-adapting in response to visual error feedback.

In both experiments, each PA session measured pointing accuracy during: baseline, adaptation, short-term (10-minutes) and long-term retention (24-hours; Fig. S1). Baseline closed- and open-loop pointing accuracy was measured in two blocks of 20 and 30 trials respectively. Adaptation comprised of alternating pairs of closed- and open-loop pointing blocks, six in Experiment 1 and seven in Experiment 2 (Fig. S1). Retention of the AE was measured 10-minutes and 24-hours after the end of PA, by means of a single block of 45 open-loop trials. In Experiment 2, 10-minute retention was followed by a washout phase in which participants pointed without wearing prisms, observed their leftward errors and therefore de-adapted. Washout consisted of 40 closed-loop trials and 45 open-loop trials distributed across six interleaved blocks (Fig. S1b). The purpose of washout was twofold. First, it enabled us to investigate whether, in the sham condition, older age was associated with a failure to de-adapt which could explain stronger AE at a later time point (see *Supplementary Results*). Second, we reasoned that, if memory formation was strengthened by stimulation during PA, then washout was more likely to interfere with long-term retention in the sham condition than in the anodal condition, which might increase sensitivity to detect the effect of stimulation at 24-hours.

### 2.3 Transcranial direct current stimulation

In Experiment 2, tDCS was delivered by a battery driven DC stimulator (Neuroconn GmbH, Ilmenau, Germany) connected to two 7 × 5 cm sponge electrodes soaked in a 0.9% saline solution. The anodal electrode was centred over C3 (5 cm lateral to Cz) corresponding to the left primary motor cortex according to the international 10–20 electrode System (Herwig et al., 2003). The cathode was placed over the right supraorbital ridge. During anodal tDCS, stimulation was applied at 1 mA for 20 min, throughout the entire adaptation phase, as in our previous work (O’Shea et al., 2017). Impedance was monitored online and kept under 10 kOhm at all time during stimulation. The current ramped up and down over a 10 s period at stimulation onset and offset. During sham tDCS, the procedure was identical except that no stimulation was delivered during the 20 min. Instead, small current pulses (110 μA over 15 ms) occurred every 550 ms to simulate the transient tingling sensations associated with real stimulation. Both experimenters and participants were blinded to the stimulation condition (anodal or sham) during behavioural testing. This was achieved by using blinding codes (“study mode” of the stimulator) provided by a researcher who was not involved in behavioural testing. Unblinding occurred at the statistical analysis stage, once data collection was completed.

In Experiment 2, participants performed two PA+tDCS sessions (anodal/sham, order counter-balanced), each separated by a minimum of one week (average interval: 10 days, *s.d.*: 6 days). This interval was chosen to allow both the effect of tDCS on cortical excitability (Nitsche, Fricke, Henschke, Schlitterlau, Liebetanz, Lang, Henning, Tergau, Paulus, 2003, Nitsche, Paulus, 2000) and the AE to wash out (O’Shea et al., 2017), to ensure a return to baseline pointing behaviour and cortical excitability by the start of the other experimental session. The rationale for stimulating during PA – as opposed to before or after – was to interact with memory formation processes occurring during exposure to the visual shift, which are known to relate to long-term retention (Inoue, Uchimura, Karibe, O’Shea, Rossetti, Kitazawa, 2015, Joiner, Smith, 2008, Kording, Tenenbaum, Shadmehr, 2007, Smith, Ghazizadeh, Shadmehr, 2006). We showed previously that M1 a-tDCS applied before – as opposed to during – PA had no effect on adaptation memory, demonstrating the importance of the interaction between neurostimulation and concurrent cognitive state (O’Shea et al., 2017).

### 2.4 MRS acquisition protocol

MRS data were acquired at the Oxford Centre for Clinical Magnetic Resonance Research (OCMR, University of Oxford), on a Siemens Trio 3-Tesla whole-body MR scanner and using a 32-channel coil. High resolution T1-weighted structural MR images (MPRAGE; 224 × 1 mm axial slices; TR/TE = 3000/4.71 ms; flip angle = 8${}^{\circ }$; FOV = 256; voxel size = 1 mm isotropic; scan time = 528 secs) were acquired for MRS voxel placement and registration purposes. MRS data were acquired from two volumes of interest (VOIs; voxel size = 2×2×2 cm^3^) in two consecutive acquisitions. The first VOI was centred on the left motor hand knob (Yousry et al., 1997) and included parts of the pre- and post- central gyrus (Fig. S2c). The second (anatomical control) VOI was centred bilaterally on the calcarine sulcus in the occipital lobe (visual cortex) (Engel, Glover, Wandell, 1997, Ip, Berrington, Hess, Parker, Emir, Bridge, 2017, Lunghi, Emir, Morrone, Bridge, 2015) (Fig. S2c). This control region was chosen because it has, to our knowledge, not been implicated in the development and/or retention of prism AEs (for review, see: Panico, Rossetti, Trojano, 2020, Petitet, O’Reilly, O’Shea, 2017). B0 shimming was performed using a GRESHIM (64 × 4.2 mm axial slices, TR = 862.56 ms, TE1/2 = 4.80/9.60 ms, flip angle = 12${}^{\circ }$, FOV = 400, scan duration = 63 secs). MR spectroscopy data (spectra) were acquired using a semi-adiabatic localization by adiabatic selective refocusing (semi-LASER) sequence (TR/TE = 4000/28 ms, 64 scan averages, scan time = 264 secs) with variable power radio frequency pulses with optimized relaxation delays (VAPOR), water suppression, and outer volume saturation (Deelchand, Adanyeguh, Emir, Nguyen, Valabregue, Henry, Mochel, Öz, 2015, Öz, Tkáč, 2011). In addition, unsuppressed water spectra were acquired from the same VOIs to remove residual eddy current effects, and to reconstruct the phased array spectra (Natt et al., 2005). Single-shot acquisitions were saved separately (single-shot acquisition mode), then frequency and phase corrected before averaging over 64 scans.

### 2.5 MRS data analysis

Metabolites were quantified using LCModel (Provencher, 2012, Provencher, 1993, Provencher, 2001) performed on all spectra within the chemical shift range 0.5 to 4.2 ppm. The model spectra were generated based on previously reported chemical shifts and coupling constants by VeSPA Project (Versatile Stimulation, Pulses and Analysis). The unsuppressed water signal acquired from the volume of interest was used to remove eddy current effects and to reconstruct the phased array spectra (Natt et al., 2005). Single scan spectra were corrected for frequency and phase variations induced by subject motion before summation. Glutamix (Glx) was used in the current study due to the inability to distinguish between glutamate and glutamine using a 3T MRI scanner. To avoid biasing the sample towards high concentration estimates, an expected relative Cramér-Rao Lower Bound (CRLB) was computed for each individual dataset given the concentration estimate and assuming a constant level of noise across all measurements (see *Supplementary Information* for detailed methods). Datasets for which the Pearson residual between the expected and observed relative CRLB exceeded 2 were excluded from subsequent analysis. Using this quality filtering criterion for γ-Aminobutyric acid (labelled GABA), Glutamix (Glutamine+Gutamate, labelled Glx) and total Creatine (Creatine + Phosphocreatine, labelled tCr), four V1 MRS datasets were discarded and no M1 MRS dataset was discarded.

Tissue correction is an important step in MRS data analysis, especially in older adults owing to brain atrophy, which has been proposed to account, at least in part, for the frequently observed age-related decline in MRS-measured GABA levels (Maes, Hermans, Pauwels, Chalavi, Leunissen, Levin, Cuypers, Peeters, Sunaert, Mantini, Puts, Edden, Swinnen, 2018, Porges, Woods, Lamb, Williamson, Cohen, Edden, Harris, 2017). LCmodel outputs metabolite concentrations for an entire volume of interest. So if the fraction of neural tissue within a volume of interest is low, owing to age-related atrophy (Good et al., 2001), metabolite concentration estimates will also necessarily be low. Several tissue correction techniques have been proposed to account for this potential confound, with currently no consensus in the literature (Harris, Puts, Edden, 2015, Maes, Hermans, Pauwels, Chalavi, Leunissen, Levin, Cuypers, Peeters, Sunaert, Mantini, Puts, Edden, Swinnen, 2018, Porges, Woods, Lamb, Williamson, Cohen, Edden, Harris, 2017). Most of these techniques make assumptions about the distribution of the metabolite of interest within the different tissue compartments. However, such assumptions may not hold across the lifespan, as the normal ageing process may affect some compartments more than others. Hence, all analyses reported in this paper used non-tissue corrected concentration estimates and instead included the percentage of grey matter (GM) and white matter (WM) in the MRS voxel as confounding variables of no interest (as in Scholl et al., 2017). Since this partial volume correction approach makes no assumption about the distribution of GABA and Glx within the different tissue types, it is particularly suitable for the present study (in which participants ranged in age from 49 to 81), and hence controls for atrophy while remaining agnostic about the differential impacts of ageing on tissue types. The percentages of grey matter, white matter, and cerebrospinal fluid present in the VOIs were calculated using FMRIB’s automated segmentation tool (Zhang et al., 2001). They are reported together with MRS data quality metrics in Table S2.

Across individuals, the total creatine (tCr) concentration estimate was negatively correlated with age in the M1 voxel ($r_{\left(21\right)}=-0.46,\;p=0.04$) although not in the V1 voxel ($r_{\left(17\right)}=-0.06,\;p=0.81$; Fig. S2b). Owing to this confound with age, tCr could not be used as a valid internal reference for metabolite estimates. Hence, throughout this work, we used absolute concentration estimates for GABA and Glx, rather than expressing the data as ratios of tCr.

### 2.6 Statistical analysis

**Fig. 1:**
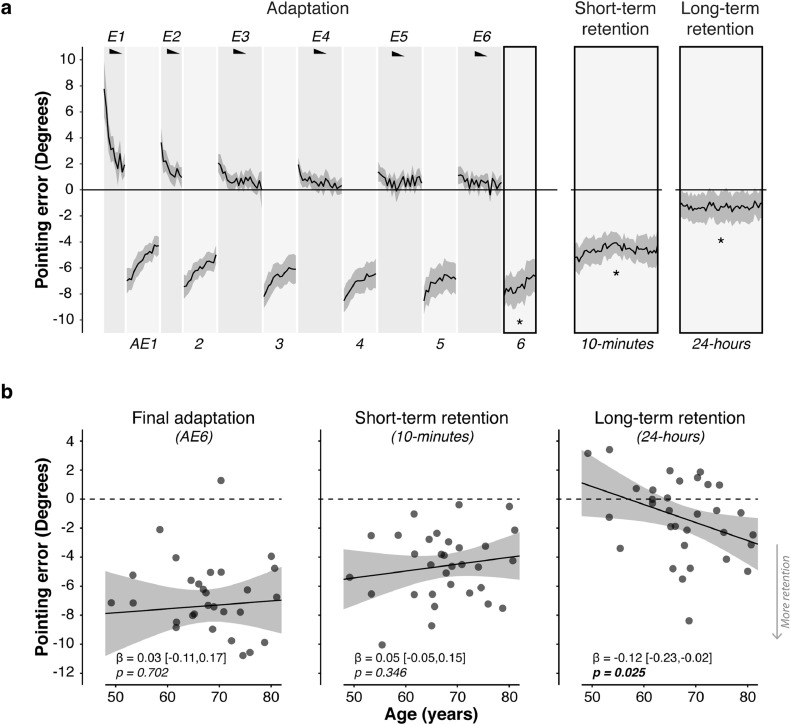
**Long-term retention of prism adaptation is higher in older adults. a.** Group mean pointing errors expressed as change from baseline accuracy (y=0). Positive y-axis values are rightward errors (i.e. in the direction of the prismatic shift), negative leftward. Error bands indicate s.e.m. Black wedges indicate blocks in which prisms were worn. During right-shifting prism exposure (E1-6), visual feedback enabled participants to correct their rightward pointing errors across trials. Consequent leftward AE was measured in intervening blocks without visual feedback throughout adaptation (AE1-6). After-effect retention was measured post-adaptation after a short (10 min) and long (24 h) interval. There was significant retention at both time points. Asterisks indicate significant one-sample t-tests for the block-averaged AE against zero (p<0.05). **b.** Age had no effect on the AE magnitude acquired by the end of adaptation (block AE6), nor on short-term retention (10 min). The key finding was that older adults showed significantly greater long-term retention (24-hours). Error bands represent the 95% Confidence Intervals. Full statistics are in Tables S3 & S4.

Statistical analyses of behaviour were performed in R (R Core Team, 2017). To control for inter-individual differences in pre-adaptation pointing accuracy, across all trials endpoint error data were normalized by subtracting the average pointing error at baseline (across left/right targets for closed-loop blocks; middle target for open-loop blocks). Unless specified otherwise, all statistical tests were two-tailed. Analyses were performed using linear regression and included checks of the following assumptions: 1) linearity, 2) homogeneity of variance, and 3) normality of residuals. These assumptions were examined visually using plots of residuals vs. observed values (linearity), fitted values vs. residuals (homogeneity of variance), and distribution of residuals (normality of residuals). Linear mixed-effects models (LMMs) were used for analyses with a longitudinal/repeated-measures component (e.g. adaptation, retention) by including intercepts and slopes as participant random effects. This approach has two advantages compared to repeated measures analyses of variance (ANOVAs): it allowed us to 1) also consider within-block behavioural dynamics, as opposed to only block average errors, and 2) dissociate random sources of inter-individual variability from meaningful ones. All model specifications are reported in Supplementary Tables. P-values were estimated using the Wald test, which corresponds to the default option of the “tab_model” function of the *sjPlot* package in R (Lüdecke, 2021). We compared LMM model parameters directly to establish neuroanatomical and neurochemical specificity. Model parameters were compared using a general linear hypothesis test using the *multcomp* package in R (Hothorn et al., 2008). For visualisation purposes, Figs. 1b, 3 and 6 b show block-averaged data as measures of retention, but the statistical analyses were run on individual trial data with random intercepts and slopes. Measures of effect size are reported for all substantial analyses, using the *effectsize* package (Ben-Shachar et al., 2020) in R. Cohen’s *d* was used to compute effect sizes for a one-sample *t*-test against zero for short- and long-term retention in Experiment 1, and for paired-samples t-tests of sham versus anodal stimulation on short- and long- term retention in Experiment 2. Approximate partial eta-squared ($\eta_{p}^{2}$) for linear mixed-effects regression analyses to summarise the proportion of variance associated with a particular fixed effect. Rules of thumb have been proposed for interpreting effect sizes. These norms for Cohen’s *d* are: small = [0.20; 0.49]; medium = [0.5; 0.79]; large ≥ 0.8. The norms for $\eta_{p}^{2}$ are: small = [0.01; 0.05]; medium = [0.06; 0.13]; large ≥ 0.14 (Cohen, 2013).

In Experiment 2, baseline OLP and CLP mean accuracy were analysed in two ways. First, to check for the absence of an order effect (PA session 1 vs. PA session 2; using pairwise t-tests). Second, to check for the absence of a stimulation condition effect (anodal tDCS session vs. sham tDCS session; using pairwise t-tests on the same data reordered by neurostimulation condition). The former analysis ensured the one week washout interval was effective (i.e. the behavioural effects of session 1 had dissipated by the onset of session 2), and the latter ensured that differences in performance between the anodal and sham tDCS conditions could be attributed to a neurostimulation effect as opposed to random systematic differences already present at baseline. To quantify the statistical evidence in favour of an absence of difference (i.e. what we aimed to achieve), a Bayes Factor ($BF_{01}$) was computed for these quality control analyses. A $BF_{01}>3$ was considered substantial evidence for the absence of difference, consistent with appropriate washout between the two experimental sessions.

Because GABA is synthesised from glutamate, the concentrations of these two neurotransmitters are typically correlated positively in the brain (Jocham et al. (2012); Stagg et al. (2011a); in our dataset, M1 GABA × M1 Glx: $r_{\left(20\right)}=0.34,\;p=0.13$; V1 GABA × V1 Glx: $r_{\left(14\right)}=0.16,\;p=0.55$). Therefore, when analysing the relationship between the absolute concentration in GABA or Glx within a voxel and outcome, the concentration of the other neurotransmitter (GABA or Glx) was also included in the model. In addition, grey and white matter concentrations were also included as covariates of no interest in all models that included neurochemical data.

A mediation analysis was used to characterise the “mechanistic” links underlying the observed correlations between age, neurochemistry, and retention. This was performed using the R package *mediation* for causal mediation analysis (Imai et al., 2010). Mediation was conducted using regression with nonparametric bootstrapping (10,000 resamples) to ascertain whether M1 inhibitory tone accounted for the link between age and long-term retention. The model included: age as the independent variable (X); absolute concentrations of M1 GABA and Glx as mediators (M_1_, M_2_); block-averaged retention at 24-hours as the dependent variable (Y) (block mean error normalised by the baseline for each individual), and controlled for the fraction of GM and WM in the M1 voxel (C_1_, C_2_). The percentage mediation ($P_{M}$) was calculated as the fraction of total effect (c) accounted by indirect effects (ab_1_ or ab_2_).

## 3 Results

### 3.1 Retention increases with age

First we tested the prediction that adaptation memory increases with age. We used a cross-sectional correlational design to measure the continuous effect of ageing across a mid- to late- life sample. This avoids the confounds inherent in a between-groups “young vs. old” design caused by gross differences in body, brain and behaviour. In Experiment 1 thirty two healthy male volunteers aged between 49 and 81 (mean age: 67.46 years, *s.d.*: 8.07; Table S1) performed a session of prism adaptation (PA) with their dominant right hand. Only men were recruited to avoid the additional variability caused by the impact of ovarian hormone fluctuations on neurotransmitter concentration in women (Epperson, Haga, Mason, Sellers, Gueorguieva, Zhang, Weiss, Rothman, Krystal, 2002, Gordon, Girdler, Meltzer-Brody, Stika, Thurston, Clark, Prairie, Moses-Kolko, Joffe, Wisner, 2015, Smith, Keel, Greenberg, Adams, Schmidt, Rubinow, Wassermann, 1999) (see *Materials and methods*).

The behavioural protocol was similar to previous work from our laboratory (Inoue, Uchimura, Karibe, O’Shea, Rossetti, Kitazawa, 2015, O’Shea, Revol, Cousijn, Near, Petitet, Jacquin-Courtois, Johansen-Berg, Rode, Rossetti, 2017) (full details in *Materials and methods*). Following PA, retention of the after-effect (AE) was assessed after a short (10-minutes) and long (24-hours) interval (Fig. S1). Effects were analysed statistically using linear mixed-effect models (LMMs) with maximal random structure. This allowed us to assess both the average angular error across task blocks and the stability of the error within blocks, while controlling for random effects of inter-individual variation.

Figure 1 a shows the pointing error data, plotted as changes from baseline (pre-adaptation) accuracy. Throughout adaptation, participants made rapid pointing movements at a $10^{\circ }$ left and right target, while wearing prism glasses that displaced their visual field $10^{\circ }$ to the right. During prism exposure (Blocks E1-6) participants gradually corrected their errors. The learning and forgetting dynamics are visible within and across blocks. At prism onset participants exhibited a large rightward error (Fig. 1a; Block E1, trial 1: mean $7.77^{\circ }$, s.e.m.: $1.05^{\circ }$, one-sample *t*-test compared to zero: $t_{\left(31\right)}=7.43,\;p<0.001$, Cohen’s *d* = 1.31) which was corrected gradually across trials and blocks (E1-6) until performance stabilized (E6) close to restored baseline accuracy (main effect of Trial within Block: $t_{\left(3185\right)}=-11.28,p<0.001$, $\eta_{p}^{2}=0.47,\;95\%CI=[0.36,0.57]$; main effect of Block: $t_{\left(3185\right)}=-9.05,p<0.001$, $\eta_{p}^{2}=0.73,\;95\%CI=[0.54,0.82]$; Table S3 - model 1).

As participants adapted gradually to the rightward visual shift, a consequent leftward AE developed, measured in interleaved blocks, critically without prisms and without visual feedback (Fig. 1a; Blocks AE1-6; mean normalised error: $-6.66^{\circ }$, $t_{\left(2865\right)}=-16.94$, p<0.001, $\eta_{p}^{2}=0.90,\;95\%CI=[0.83,0.94]$; Table S3 - model 2). This prism AE is the key experimental measure. On AE trials, the absence of visual feedback prevents error-based learning and requires participants to rely on internal representations of sensed limb position to guide their movements. Thus, the leftward AE expresses the visuomotor transformation acquired during prism exposure. Its persistence after prism removal is the measure of adaptation memory. The AE was measured after each block of prism exposure (AE1-6, Fig. S1). Initially memory was labile: on the first trial of the first block the AE was large ($-6.99^{\circ }$), but across the 15 trials of the first block it decayed by $2.70^{\circ }$ on average. Subsequent blocks of prism exposure led the AE to gradually stabilize, evidenced by the progressive flattening of slopes across blocks AE1-6 (interaction Trial × Block: $t_{\left(2865\right)}=-3.33$, p=0.001, $\eta_{p}^{2}=0.26,\;95\%CI=[0.04,0.48]$; Fig. 1a; Table S3 - model 2). Thus, our protocol induced an adaptation memory trace that consolidated gradually across the Adaptation phase.

The critical measure of memory was AE retention post-adaptation (Fig. 1a-b). After 10 min of blindfolded rest, there was significant short-term retention (mean error: $-4.61^{\circ }$, s.e.m.: $0.41^{\circ }$, $t_{\left(1434\right)}=-11.36$, p<0.001; one sample *t*-test of mean retention: $t_{\left(31\right)}=-11.18$, p<0.001, Cohen’s *d* = -1.98, 95%CI = [-2.61, -1.39]; Table S3 - model 3). Long-term retention, measured 24 h later, was also significant (mean error: $-1.30^{\circ }$, s.e.m.: $0.48^{\circ }$, $t_{\left(1434\right)}=-2.75$, p=0.006, $\eta_{p}^{2}=0.19,\;95\%CI=[0.01,0.42]$; one sample *t*-test of mean retention: $t_{\left(31\right)}=-2.70$, p=0.01, Cohen’s *d* = -0.48, 95%CI = [-0.85, -0.11]; Table S3 - model 4). The AE was stable at both time points, indicated by no change in error across trials (main effect of Trial: both p>0.38).

Our hypothesis was that AE retention would increase with older age. To avoid inflating the risk of Type 1 errors and focus on the testing of this *a priori* hypothesis, our analysis was restricted to the AE post-adaptation. For completeness, the association between age and adaptation behaviour is reported in *Supplementary Results*. Age had no significant effect on the AE magnitude acquired by the end of prism exposure (Block AE6), nor on short-term retention (both p>0.35; Fig. 1b; Table S4 - models 1 & 2). However, older age was associated with greater long-term retention (Age × AE_24hrs_: $t_{\left(1432\right)}=-2.24,\;p=0.025$, $\eta_{p}^{2}=0.14,\;95\%CI=[0.00,0.36]$, Fig. 1b, Table S4 - model 3). This association remained significant when controlling for the AE at the two preceding time points (AE6 or 10-min retention), and when controlling for average reaching speed during prism exposure (slower movements, expected in ageing, could arguably favour retention; Table S4 - models 4–6). Moreover, the analysis of de-adaptation data from the sham condition in Experiment 2 revealed that the relationship between age and long-term retention was not due to an inability to recover normal accuracy during de-adaptation. Instead, older age was associated with a longer-lasting tendency to spontaneously re-express an AE when deprived of visual feedback, a behaviour that reflects enhanced adaptation memory (see *Supplementary Results*).

### 3.2 Motor cortical inhibitory tone declines with age

Next, we tested for an expected decrease in motor cortical inhibitory tone with older age. Three Tesla magnetic resonance spectroscopy was used to quantify neurochemical concentration in left sensorimotor cortex (labelled “M1”), and in a control region of occipital cortex (labelled “V1”; see *Materials and methods*; Fig. S2). The metabolites of interest were GABA and Glutamix (“Glx” = Glutamate + Glutamine, since these two metabolites cannot be distinguished reliably at 3 Tesla). Unsurprisingly, in both regions, age was associated with significant grey matter atrophy (both p<0.002), which could indirectly lower neurochemical concentration estimates. Hence, all analyses of neurochemistry ruled out this potential confound by controlling for grey and white matter fractions within each region using multiple regression (see *Materials and methods*). To minimize multiple comparisons, analyses focused on the ratio of excitation:inhibition (E:I = Glx:GABA). If an effect was significant, follow-up analyses assessed the individual contributions of Glx and GABA. Note that the E:I metric used throughout this study refers solely to MRS-derived neurochemical concentration measures. There may be other factors influencing the net E:I (e.g. receptor concentration or effectiveness) that may not be captured by this measure (Cuypers, Hehl, van Aalst, Chalavi, Mikkelsen, Van Laere, Dupont, Mantini, Swinnen, 2021, Stagg, Bestmann, Constantinescu, Moreno Moreno, Allman, Mekle, Woolrich, Near, Johansen-Berg, Rothwell, 2011).

**Fig. 2:**
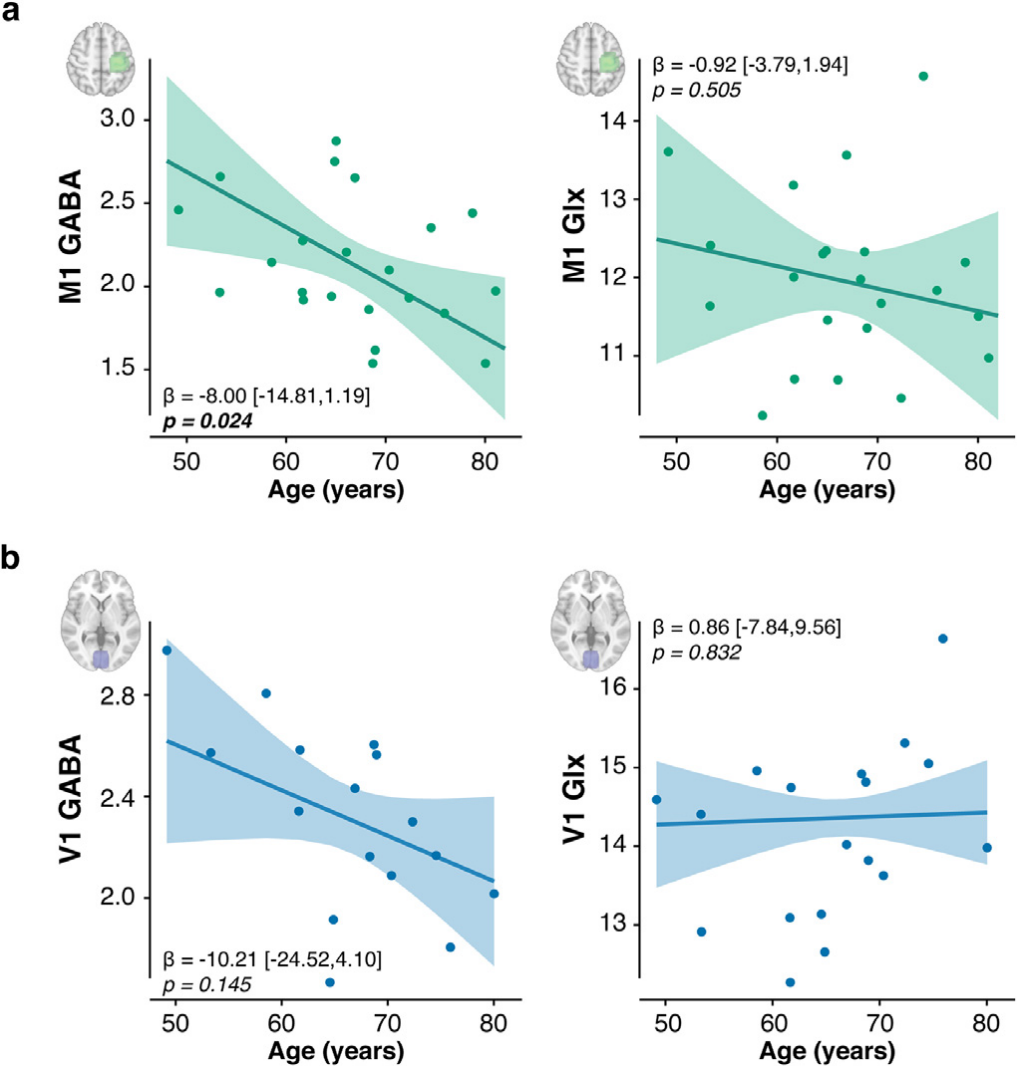
**Motor cortical inhibitory tone is lower in older adults. a.** The concentration of GABA but not Glutamix (Glutamate + Glutamine, Glx) was associated negatively with age in the left sensorimotor cortex (labelled "M1"). **b.** There was no significant association between age and neurochemical concentration in occipital cortex (labelled "V1"). For each voxel and neurotransmitter, plotted relationships control for the fraction of grey matter and white matter, and the other neurotransmitter. Absolute concentrations are expressed in arbitrary units. Error bands represent the 95% Confidence Intervals. Full statistical details are in Table S5.

Multiple linear regressions showed that sensorimotor cortex E:I increased with age (standardised $\beta_{age}=0.66$, $t_{\left(18\right)}=2.09$, p=0.051, $\eta_{p}^{2}=0.10,\;95\%CI=[0.00,0.39]$; Table S5 - model 1). As predicted, across individuals, as age increased, M1 GABA concentration decreased (standardised $\beta_{age}=-0.74$, $t_{\left(17\right)}=-2.48$, p=0.024, $\eta_{p}^{2}=0.14,\;95\%CI=[0.00,0.45]$; Table S5 - model 2). There was no such relationship with Glx (standardised $\beta_{age}=-0.23$, $t_{\left(17\right)}=-0.68$, p=0.51, $\eta_{p}^{2}=0.02,\;95\%CI=[0.00,0.29]$; Fig. 2a, Table S5 - model 3).

In the anatomical control region (occipital cortex), there was a qualitatively similar pattern of age-related inhibitory decline, consistent with previous reports (Chamberlain, Gagnon, Lalwani, Cassady, Simmonite, Seidler, Taylor, Weissman, Park, Polk, 2021, Simmonite, Carp, Foerster, Ossher, Petrou, Weissman, Polk, 2019). However this was not statistically significant (Age × V1 E:I: standardised $\beta_{age}=0.39$, $t_{\left(12\right)}=1.46$, p=0.171, $\eta_{p}^{2}=0.46,\;95\%CI=[0.05,0.71]$ ; Table S5 - model 4), likely reflecting the impact of quality controls that reduced the size of the occipital dataset, and consequently reduced power (Table S1 & S2). Even when decomposing the E:I into its GABA and Glx constituents, none of these metabolites showed a significant relationship with age in V1 (Age × V1 GABA, controlling for V1 Glx: standardised $\beta_{age}=-0.40$, $t_{\left(11\right)}=-1.57$, p=0.145, $\eta_{p}^{2}=0.51,\;95\%CI=[0.07,0.75]$; Age × V1 Glx, controlling for V1 GABA: standardised $\beta_{age}=0.04$, $t_{\left(11\right)}=0.22$, p=0.832, $\eta_{p}^{2}=0.20,\;95\%CI=[0.00,0.56]$; Table S5 - models 5 & 6).

### 3.3 Lower motor cortical inhibitory tone is associated with greater long‐term retention

**Fig. 3:**
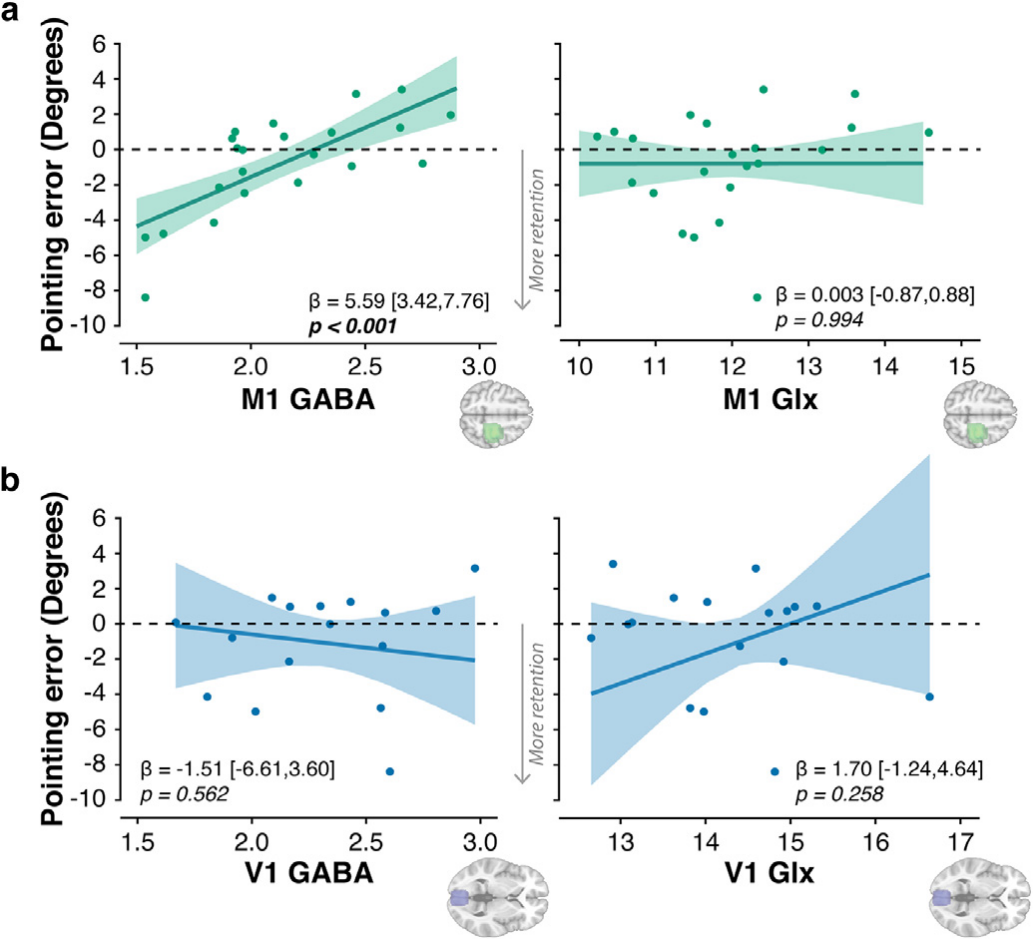
**Lower motor cortical inhibitory tone is associated with greater long-term retention.** Plot shows relationships between brain chemistry and the magnitude of prism after-effect retained 24 h after adaptation. Negative values on the y-axis indicate retention. **a. Sensorimotor cortex ("M1")** Across individuals, lower GABA was associated with greater retention. There was no relationship with Glx (Glutamate + Glutamine). **b. Occipital cortex (**"**V1")** There was no relationship between GABA or Glx and 24-hour retention. For each voxel and neurotransmitter, relationships control for the fraction of grey matter and white matter, and the other neurotransmitter. Absolute concentrations are expressed in arbitrary units. Error bands represent the 95% Confidence Intervals. Full statistics details are in Table S6.

Based on our previous work (O’Shea et al., 2017), we hypothesized that lower motor cortical inhibitory tone would be associated with greater retention. Results confirmed this prediction (Fig. 3). Across individuals, higher sensorimotor cortex E:I was associated with a larger prism AE at retention 24-hours after adaptation ($t_{\left(980\right)}=-5.40$, p<0.001, $\eta_{p}^{2}=0.58,\;95\%CI=[0.27,0.74]$; Table S6 - model 1). A follow-up LMM revealed that this relationship was driven by GABA: individuals with lower M1 GABA concentration showed greater retention ($t_{\left(978\right)}=5.04$, p<0.001, $\eta_{p}^{2}=0.55,\;95\%CI=[0.23,0.73]$; Fig. 3a, Table S6 - model 2). There was no such relationship with M1 Glx ($t_{\left(978\right)}=0.01,p=0.99$, $\eta_{p}^{2}<0.001,\;95\%CI=[0.00,0.00]$; Fig. 3a, Table S6 - model 2). Thus, this memory effect was neurochemically specific (M1 GABA vs. M1 Glx: z=3.56, p<0.001). It was also anatomically specific (M1 GABA vs. V1 GABA: z=2.80, p=0.005): there was no relationship between retention and V1 metabolites - not for GABA, Glx or E:I (all p>0.25; Fig. 3b, Table S6 - models 5 & 6). As before, the results were unchanged when controlling for average movement time during prism exposure (Table S6 - models 3, 4, 7, 8).

### 3.4 Retention increases with age as a function of motor cortical inhibitory decline

Our key prediction was that as M1 GABA concentration declines with age, adaptation memory would increase, and the former would explain the latter. We used mediation analysis to formally test this hypothesis. Mediation analysis is well suited to a situation in which the independent variable (Age) may not directly influence the dependent variable (Long-term retention), but is instead hypothesized to do so indirectly via its effect on candidate mediators (M1 E:I, GABA, Glx). The extent to which the relationship between the independent and dependent variable is influenced by a mediator is termed the indirect effect. We tested the significance of indirect effects using a bootstrap estimation approach with 10,000 samples (see *Materials and methods*).

**Fig. 4:**
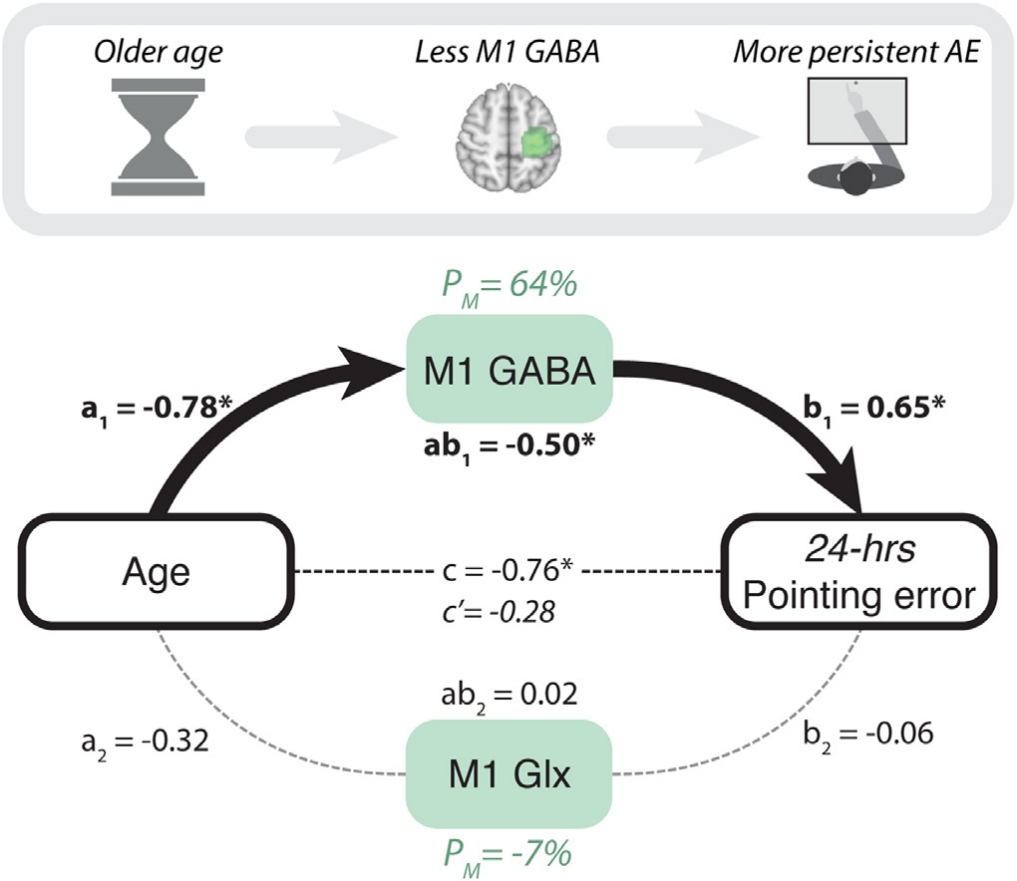
**Adaptation memory is stronger in older age owing to the decline in motor cortical inhibitory tone.** A mediation model tested whether M1 neurochemistry explained the relationship between age and retention. Consistent with our mechanistic hypothesis, GABA, but not Glx, mediated the positive relationship between age and 24-hour retention, explaining 64% of the variance. Standardised regression coefficients are reported next to the corresponding paths. Asterisks indicate significance (p<0.05). Full statistics: Table 6. Independent variable: Age. Dependent variable: AE 24-hours post-adaptation. Mediators: M1 GABA and Glx (controlling for grey and white matter tissue fractions).

Figure 4 shows that, as hypothesized, the effect of age on long-term retention was mediated by motor cortical E:I ($ab_{1}=-0.41$, 95%CI: [−1.45,−0.08], p=0.017). More specifically, the indirect effect was driven by M1 GABA and not Glx. M1 GABA was a significant mediator ($ab_{1}=-0.50$, 95%CI: [−1.46,−0.16], p=0.0086), accounting for 64% of the variance between age and long-term retention (Fig. 4, Table 6), while M1 Glx showed no such effect ($ab_{2}=0.018$, 95%CI: [−0.095,0.31], p=0.74). When M1 neurochemistry was controlled for, age was no longer a significant predictor of 24-hour retention ($c^{\prime }=-0.28,\;p=0.38$), consistent with full mediation. Thus, age-related decline in sensorimotor GABA explains stronger adaptation memory in older age. Once again, results were unchanged when controlling for average movement time during prism exposure (Table 6).

### 3.5 How stimulation changes memory depends on motor cortical E:I

The mediation model indicated that the M1 GABA decline was responsible for the memory increase in older adults. However, the cross-sectional study design precludes direct causal inference (Marinescu et al., 2018). Hence, to more directly test causation, we intervened experimentally with anodal transcranial direct current stimulation (a-tDCS) during PA. M1 a-tDCS has been shown to increase motor cortical E:I in young (Bachtiar, Near, Johansen-Berg, Stagg, 2015, Barron, Vogels, Emir, Makin, O’Shea, Clare, Jbabdi, Dolan, Behrens, 2016, Kim, Stephenson, Morris, Jackson, 2014, Patel, Romanzetti, Pellicano, Nitsche, Reetz, Binkofski, 2019, Stagg, Best, Stephenson, O’Shea, Wylezinska, Kincses, Morris, Matthews, Johansen-Berg, 2009) and older (Antonenko et al., 2017) adults. In addition, we previously showed (in young adults) that M1 a-tDCS during PA increased short- and long- term retention, in proportion to the stimulation-induced E:I increase (O’Shea et al., 2017). However, given our finding in Experiment 1 (Fig. 2) – that M1 E:I is already naturally high in older age – we expected there may be a ceiling effect on further increasing E:I in some individuals. Hence, if M1 E:I causally mediates adaptation memory, individuals with naturally low M1 E:I should benefit from excitatory a-tDCS, and show increased adaptation memory. By contrast, in older individuals their naturally elevated M1 E:I may leave little to no room for a further excitability increase and consequent memory gain from M1 a-tDCS. We therefore predicted a positive relationship between baseline M1 E:I and the stimulation-induced change in prism AE at 24-hours (negative $\Delta AE_{24hrs}$ indicates enhanced retention, positive $\Delta AE_{24hrs}$ indicates impaired retention).

To test this hypothesis, a sub-set of twenty-five participants from Experiment 1 (mean age: 69.6 years, *s.d.*: 8.4; Table S1) consented to undergo a follow-up study (Experiment 2), in which tDCS (anodal/sham, counterbalanced repeated measures design) was applied in two weekly test sessions to left M1 during adaptation, and retention was assessed after 10 min and 24 h (see *Materials and methods*, Fig. S1). A quality control analysis showed that pointing accuracy was not statistically different at baseline between the first and second experimental sessions (pairwise t-tests; baseline OLP: $t_{\left(24\right)}=0.04$, p=0.97, $BF_{01}=4.74$; baseline CLP: $t_{\left(24\right)}=-1.13$, p=0.27, $BF_{01}=2.68$), or between the anodal and sham experimental sessions (pairwise t-tests; baseline OLP: $t_{\left(24\right)}=0.49$, p=0.63, $BF_{01}=4.26$; baseline CLP: $t_{\left(24\right)}=0.61$, p=0.55, $BF_{01}=4.01$).

**Fig. 5:**
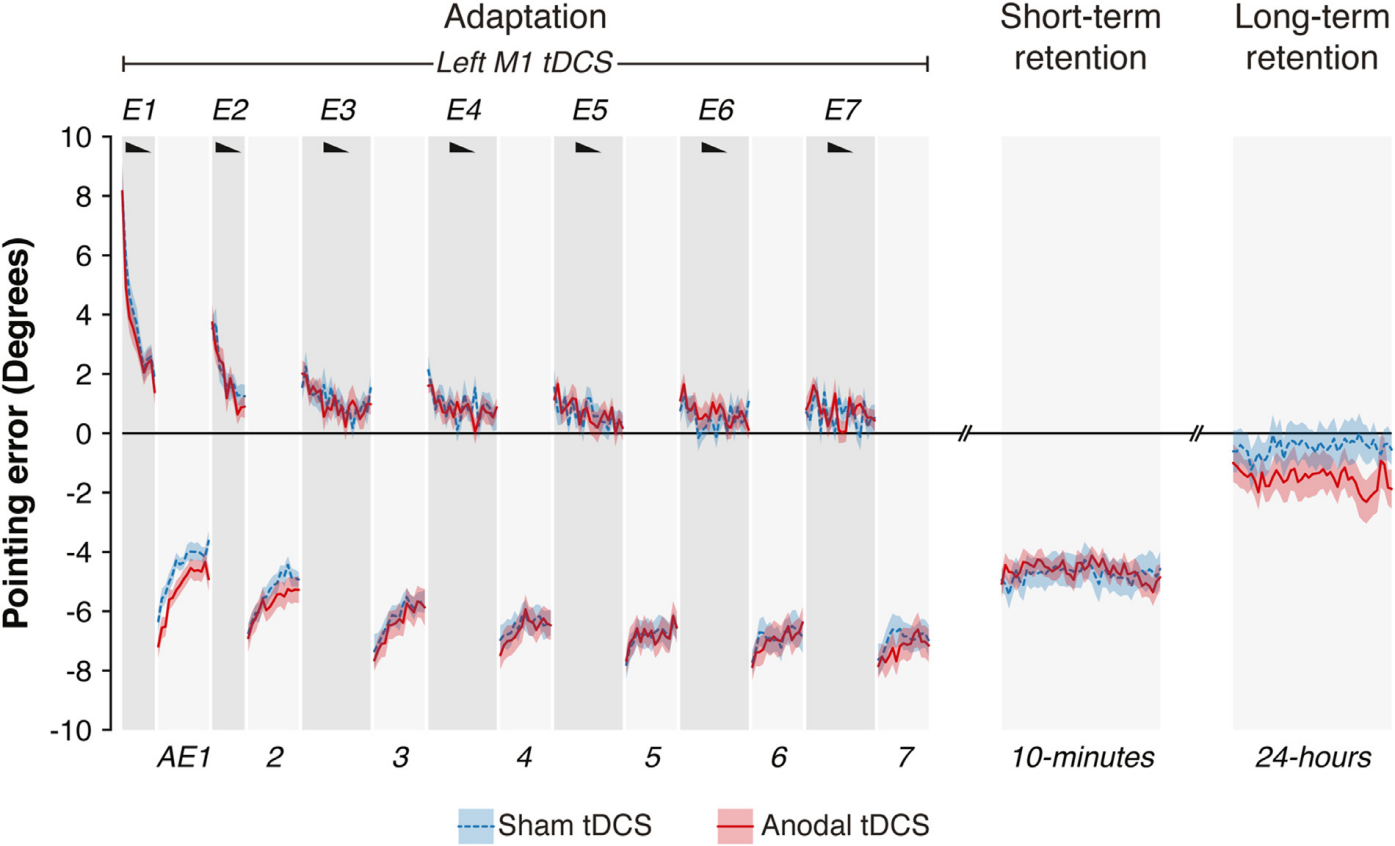
**On average across older adults excitatory stimulation of M1 during adaptation did not increase retention.** Timecourse of pointing errors for the same behavioural paradigm and graph conventions as in Fig. 1, except that stimulation (anodal or sham tDCS) was applied to left M1 throughout the adaptation phase. Errors are normalised to baseline (pre-adaptation) accuracy. Negative values on the y-axis indicate prism after-effects. Error bands indicate s.e.m. After excitatory stimulation of M1 during adaptation, retention increased numerically but not significantly, contrary to our previous findings in young adults, but consistent with our expectations in this cohort of older adults.

Figure 5 shows the group-averaged normalised pointing data. Stimulation had no effect on short-term retention ($t_{\left(2235\right)}=0.22$, p=0.83, $\eta_{p}^{2}=0.002,\;95\%CI=[0.00,0.14]$; paired-samples *t*-test of mean retention, anodal vs sham: $t_{\left(24\right)}=0.21$, p=0.83, Cohen’s *d* = 0.04, 95%CI = [-0.36, 0.44]). Although long-term retention increased numerically, this was not statistically significant ($t_{\left(2235\right)}=-1.35$, p=0.18, $\eta_{p}^{2}=0.07,\;95\%CI=[0.00,0.31]$; paired-samples *t*-test of mean retention, anodal vs sham: $t_{\left(24\right)}=-1.32$, p=0.20, Cohen’s *d* = -0.26, 95%CI = [-0.67, 0.14]; Table S7 - model 1). The lack of a significant memory gain from stimulation across the group contrasts with our previous findings in young adults (O’Shea et al., 2017).

**Fig. 6:**
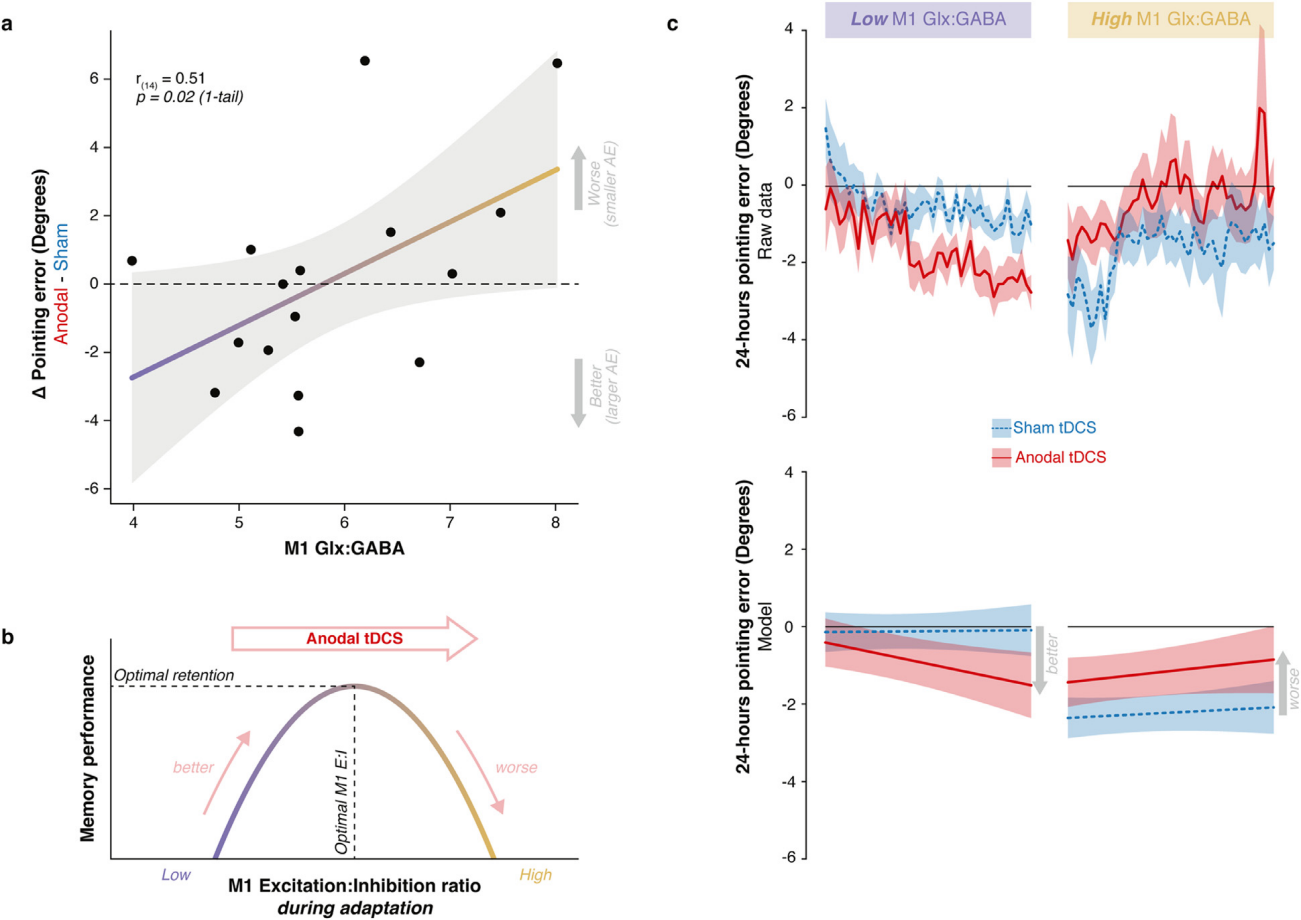
**How stimulation changes memory depends on motor cortical E:I. a.** Individuals’ M1 E:I (Glx:GABA) is plotted against the stimulation effect (anodal - sham difference in normalised pointing error at 24-hour retention). On the y-axis, negative values indicate greater retention with anodal tDCS compared to sham. Positive values indicate the opposite. Across individuals, stimulation enhanced retention in those with low E:I and impaired retention in those with high E:I. These data confirm the hypothesis that retention depends causally on M1 E:I. **b.** The schematic offers an explanatory account of the data in panel a. Under the assumption that stimulation increases E:I across the group, the distribution of induced memory change has an inverted U-shape. This suggests there is an optimal range of E:I within which retention is maximal. The optimum differs across individuals. By increasing E:I, stimulation moves individuals with low E:I towards maximum, increasing retention. But for individuals with high E:I, who are close to maximum, stimulation exceeds the optimum and so retention becomes impaired. **c.** A moderation analysis confirmed that how stimulation changed memory varied as a function of M1 E:I (Glx:GABA × tDCS : $t_{1419}=2.40$, p=0.017). For visualisation purposes, this interaction is illustrated using a median split on the M1 E:I data. The data (top) and model fit (bottom) are plotted separately for individuals with low versus high M1 E:I, and show opposing effects of excitatory stimulation on adaptation memory that depend on individuals’ M1 E:I. Error bands in panel a represent the 95% Confidence Intervals; error bands in panels c and d represent standard error of the mean.

To test the key hypothesis, that individual differences in intrinsic motor cortical E:I would influence the magnitude of stimulation-induced memory change, we conducted a moderation analysis. For all participants who had undergone a MRS scan in Experiment 1 (n=16; Table S1) we added their M1 Glx:GABA levels from that scan to the linear mixed model analyses of the effect of stimulation on retention. As predicted, the effect of stimulation on long-term retention interacted significantly with motor cortical E:I (E:I × a-tDCS: $t_{\left(1419\right)}=2.40$, p=0.009, one-tail, $\eta_{p}^{2}=0.26$; Fig. 6; Table S7 - model 2).

Figure 6 plots the result of the moderation analysis for long-term retention. Panel a shows how the induced memory change varied as a function of M1 E:I – in those individuals with low E:I, stimulation enhanced retention; in individuals with high E:I, stimulation impaired retention. Panel b offers an explanatory account. Under the assumption that M1 a-tDCS increases E:I (Antonenko, Schubert, Bohm, Ittermann, Aydin, Hayek, Grittner, Flöel, 2017, Bachtiar, Near, Johansen-Berg, Stagg, 2015, Barron, Vogels, Emir, Makin, O’Shea, Clare, Jbabdi, Dolan, Behrens, 2016, Kim, Stephenson, Morris, Jackson, 2014, Patel, Romanzetti, Pellicano, Nitsche, Reetz, Binkofski, 2019, Stagg, Best, Stephenson, O’Shea, Wylezinska, Kincses, Morris, Matthews, Johansen-Berg, 2009), the pattern of induced memory change followed an inverted U-shaped distribution, which suggests there is an optimum level of E:I at which retention is maximal. Increasing E:I via stimulation enhanced memory in those with low E:I, up to an optimum level beyond which stimulation had a deleterious effect, impairing retention. Panel c illustrates the result of the moderation analysis via a median split on the M1 E:I data.

A follow-up LMM decomposed the E:I data to assess the moderating roles of M1 GABA and Glx separately. Both Glx (Glx × a-tDCS: $t_{\left(1415\right)}=2.57$, p=0.005, one-tail, $\eta_{p}^{2}=0.29,\;95\%CI=[0.01,0.58]$) and GABA (GABA × a-tDCS: $t_{\left(1415\right)}=-1.73$, p=0.042, one-tail, $\eta_{p}^{2}=0.16,\;95\%CI=[0.00,0.47]$) moderated the stimulation effect, each in opposite directions (Table S7 - model 3). Across individuals, stimulation increased retention in those with higher GABA and/or lower Glx, and impaired retention in those with lower GABA and/or higher Glx. This result was not observed for V1 neurochemistry (Table S7 - models 4 & 5). Controlling for the magnitude of the AE reached at the end of prism exposure (AE6) or at the 10-min retention time point did not change the results.

Given the finding in Experiment 1 of an association between age and M1 E:I (Fig. 2), we tested whether age could be substituted as a simpler, easy to measure, proxy for neurochemistry. By contrast with M1 E:I, age alone did not moderate the effect of stimulation on retention (see *Supplementary Results*; Fig. S3), reinforcing the idea that it is indeed M1 neurochemistry – and not age *per se* – that is critical to determining adaptation memory.

## 4 Discussion

This study tested the hypothesis that healthy older adults would exhibit stronger adaptation memory, owing to age-related M1 GABA decline. The results confirmed this prediction. Within a cross-sectional sample of healthy men (aged 49–81 years), older age was associated with higher long-term retention (Fig. 1) and lower M1 GABA (Fig. 2). A mediation analysis showed that the latter explained the former (Figs. 3 & 4). When M1 neurochemistry was accounted for, there was no longer a relationship between age and memory, consistent with full mediation. The findings were specific: anatomically (M1 not V1) and neurochemically (GABA not Glx). To more directly infer a causal link between neurochemistry and memory, anodal tDCS was used to experimentally lower M1 inhibitory tone (Antonenko, Schubert, Bohm, Ittermann, Aydin, Hayek, Grittner, Flöel, 2017, Barron, Vogels, Emir, Makin, O’Shea, Clare, Jbabdi, Dolan, Behrens, 2016, Stagg, Best, Stephenson, O’Shea, Wylezinska, Kincses, Morris, Matthews, Johansen-Berg, 2009) (thus increasing E:I) in a subset of the same participants. Individuals’ intrinsic E:I ratio within sensorimotor cortex moderated how stimulation affected memory (Fig. 6). In those with naturally low E:I (low Glx and/or high GABA) stimulation increased retention, whereas in those with naturally high E:I (high Glx and/or low GABA) stimulation impaired retention. The distribution of stimulation-induced memory change was consistent with an inverted U-shape, suggesting there is an optimum range of M1 E:I within which adaptation memory is maximal. Once again, the results were specific to E:I within M1 (no effect for V1). Whereas GABA loss in older age has typically been associated with functional decline (Heise, Zimerman, Hoppe, Gerloff, Wegscheider, Hummel, 2013, Hermans, Leunissen, Pauwels, Cuypers, Peeters, Puts, Edden, Swinnen, 2018, King, Rumpf, Verbaanderd, Heise, Dolfen, Sunaert, Doyon, Classen, Mantini, Puts, Edden, Albouy, Swinnen, 2020, Papegaaij, Taube, Hogenhout, Baudry, Hortobágyi, 2014, Swanson, Fling, 2018), the present results reveal a specific domain of motor function that instead becomes naturally potentiated. Enhanced adaptation memory may help compensate for impaired motor skill learning in older age (Roig et al., 2014).

In our study, the joint effect of age and M1 GABAergic inhibition was restricted to open-loop pointing and the 24-hours retention time-point. It could therefore be argued that the age-associated larger after-effect (AE) at 24-hours may reflect a strategic inability to apply the appropriate visuomotor mapping to the task context (i.e. older participants fail to de-adapt and instead persist with pointing as though still perturbed by prisms, even though they were removed 24 h earlier). By this account, older individuals with higher M1 E:I do not have stronger retention, but instead have a deficit in contextual switching. We think this is unlikely for two reasons. First, use of a cognitive strategy – which is thought to decline with normal ageing (Vandevoorde and Orban de Xivry, 2019) – is known to contribute little to the prism AE (Redding et al., 1992). Instead, the magnitude of the AE on open-loop pointing is thought to reflect automatic sensorimotor realignment processes that evolve rather independently from strategic control (Panico, Rossetti, Trojano, 2020, Petitet, O’Reilly, O’Shea, 2017, Redding, Rossetti, Wallace, 2005). Second, if ageing was merely associated with an inability to switch strategy between task contexts, its effect should be equally manifest throughout the entire experiment, or be most pronounced early on, when participants first learn to task switch and do so frequently (i.e. every 3 min over a 20-minute period, when alternating between CLP and OLP blocks during adaptation). However, in our data, there was no effect of age on OLP during adaptation. Instead, the effect of age was constrained to the late retention time point 24 h post-adaptation (Fig. 1), and unfolded gradually during active washout (see *Supplementary Results*). Hence, in our view, the more parsimonious interpretation of these results is that older adults with higher E:I have stronger adaptation memory rather than a deficit in contextual switching. This of course does not rule out 24-hours retention also being influenced by other processes that occur during the post-adaptation interval that may contribute to the effects observed here.

The current work provides strong evidence at the level of individuals that differences in adaptation memory relate to differences in sensorimotor neurochemistry. This need not be interpreted as evidence that memories are formed and/or stored locally and/or exclusively in sensorimotor cortex. Of note, we measured brain chemistry only in M1 and V1. Adaptation memory, like most functions, is likely to be distributed, implemented through parieto-premotor-cerebellar circuit interactions. Yet we targeted M1 owing to evidence that it has a causal role in the early consolidation of motor learning (Hadipour-Niktarash, Lee, Desmond, Shadmehr, 2007, Hunter, Sacco, Nitsche, Turner, 2009, Landi, Baguear, Della-Maggiore, 2011, Li, Padoa-Schioppa, Bizzi, 2001, Richardson, Overduin, Valero-Cabré, Padoa-Schioppa, Pascual-Leone, Bizzi, Press, 2006). These data strengthen this evidence base in the case of adaptation. We interpret the data to indicate that M1 is a privileged node in the distributed cortical circuitry that implements the early formation of adaptation memory. That is, the strength of that memory trace can be changed during its formation by tonic disinhibition of M1 (via a-tDCS), and the impact on individuals’ memory is quantitatively related to their local E:I balance within M1. This local neurochemical measure has been shown to correlate with sensorimotor network resting state functional connectivity (Antonenko, Schubert, Bohm, Ittermann, Aydin, Hayek, Grittner, Flöel, 2017, Bachtiar, Near, Johansen-Berg, Stagg, 2015, Stagg, Bachtiar, Amadi, Gudberg, Ilie, Sampaio-Baptista, O’Shea, Woolrich, Smith, Filippini, Near, Johansen-Berg, 2014). Thus, M1 E:I may be an informative measure because it also serves as a proxy readout of sensorimotor network strength. Extra-synaptic GABAergic tone, that measured by magnetic resonance spectroscopy, has also been linked to oscillatory markers of inter-regional neuronal communication (Buzsáki, Schomburg, 2015, Muthukumaraswamy, Edden, Jones, Swettenham, Singh, 2009). Hence, this local M1 readout may also indirectly index inter-individual differences in propensity for inter-areal communication strength, of functional relevance during adaptation. Thus, we conclude that M1 is a sensitive node at which to both measure and manipulate adaptation memory formation.

Two manipulations, one natural – ageing (Experiment 1; Fig. 4) – and one experimental – brain stimulation (Experiment 2; Fig. 6) – indicate that adaptation memory depends causally on M1 E:I. Collectively, they show that, on average, lower inhibitory tone is associated with stronger retention. However, the inverted U-shaped response to neurostimulation (Fig. 6) suggests that there may be an optimal level of E:I at which retention is maximal. Increasing E:I via stimulation moves individuals with naturally low E:I towards this maximum, whereas individuals with naturally high E:I may exceed that maximum and retention becomes impaired. On average, relatively younger adults are more likely to have E:I levels below this theoretical upper physiological bound, while older adults are closer to it. This may explain the absence of a significant overall group mean memory enhancement effect of stimulation in the present sample of older adults (Fig. 5), by contrast with our previous findings in young adults (O’Shea et al., 2017). This suggests that, in general, inducing plasticity with M1 a-tDCS is, on average, likely to be less effective in older adults – at least to the extent to which it depends on lowering inhibitory tone (Antonenko, Schubert, Bohm, Ittermann, Aydin, Hayek, Grittner, Flöel, 2017, Zimerman, Nitsch, Giraux, Gerloff, Cohen, Hummel, 2013). Nonetheless, age *per se* did not predict response to stimulation (Fig. S3), whereas M1 E:I did, underlining the potential utility of M1 Glx/GABA as biomarkers of inter-individual variation in stimulation response. The present results reveal that adaptation memory and M1 a-tDCS effects share a common neurochemical substrate: causal dependence on M1 inhibitory tone. This mechanistic synergy makes M1 a-tDCS a particularly suitable manipulation for understanding retention of adaptation, and vice versa.

An alternative interpretation of the dependency of the behavioural effect of stimulation on baseline neurochemistry (to that offered above, Fig. 6b) is that M1 a-tDCS may have *reduced*, rather than increased, E:I in individuals with naturally high E:I, contrary to the effect predominantly reported in the literature (Antonenko, Schubert, Bohm, Ittermann, Aydin, Hayek, Grittner, Flöel, 2017, Barron, Vogels, Emir, Makin, O’Shea, Clare, Jbabdi, Dolan, Behrens, 2016, Kim, Stephenson, Morris, Jackson, 2014, Stagg, Best, Stephenson, O’Shea, Wylezinska, Kincses, Morris, Matthews, Johansen-Berg, 2009). If, in those individuals with high M1 E:I, excitation is already near physiological ceiling, then anodal stimulation might trigger homeostatic regulatory mechanisms (that protect against over-excitation) to instead reduce E:I (Karabanov, Ziemann, Hamada, George, Quartarone, Classen, Massimini, Rothwell, Siebner, 2015, Krause, Márquez-Ruiz, Kadosh, 2013, Lang, Siebner, Ernst, Nitsche, Paulus, Lemon, Rothwell, 2004, Nitsche, Roth, Kuo, Fischer, Liebetanz, Lang, Tergau, Paulus, 2007, Siebner, Lang, Rizzo, Nitsche, Paulus, Lemon, Rothwell, 2004) (Fig. S4). Thus, impaired retention would not be explained by the falling part of the inverted U-shaped [retention × M1 E:I] function that we propose in Fig. 6b. Rather, it would arise from E:I rebound via homeostasis when stimulation causes the excitability ceiling to become breached (Fig. S4). The relative (non-exclusive) contributions of these two potential mechanisms to impaired retention is a question for future work. Nonetheless, under either scenario, the data are consistent with the existence of an optimal range of M1 E:I within which retention is maximal.

Interestingly, our findings suggest that, in those with naturally high baseline E:I, priming M1 with inhibitory stimulation (e.g. using preconditioning cathodal tDCS or low-frequency repetitive Transcranial Magnetic Stimulation; Lang, Siebner, Ernst, Nitsche, Paulus, Lemon, Rothwell, 2004, Siebner, Lang, Rizzo, Nitsche, Paulus, Lemon, Rothwell, 2004) might offer a way to increase their sensitivity to the effect of subsequent excitatory stimulation, and thus induce increased retention. This type of strategy has been used elsewhere (Fujiyama, Hinder, Barzideh, Van de Vijver, Badache, Manrique-C, Reissig, Zhang, Levin, Summers, Swinnen, 2017, Pourmajidian, Lauber, Sidhu, 2020) and may be particularly suited to the present paradigm.

The current results extend our previous behavioural findings that M1 a-tDCS during adaptation boosts therapeutic efficacy in post-stroke visual neglect (O’Shea et al., 2017). The present data support the idea that M1 a-tDCS might in fact antagonize (rather than enhance) PA therapy in some patients. Stroke disrupts E:I across distributed cortical networks, and how this interacts with age-related dysregulation is likely to vary by region and time. How the neurochemical constraints identified here apply in stroke populations remains to be tested. The present normative dataset could help guide interpretation of future stroke data. Serendipitously, in our earlier work, we established proof of concept via experiments in young adults (O’Shea et al., 2017). These showed that M1 a-tDCS during adaptation specifically enhanced retention. If instead we had started by testing older healthy controls, we are unlikely to have ever progressed to testing prism therapy + M1 a-tDCS in neglect, since (as in Fig. 5) we would not have found evidence that stimulation enhances retention. What reconciles our previous and present results, across the combined evidence from younger and older adults, is the new physiological insight that individuals’ motor cortical E:I – both intrinsic and induced – governs this form of motor memory plasticity.

Previous work has generally found adaptation (error-based learning) to be preserved or somewhat impaired in older adults (Anguera, Reuter-Lorenz, Willingham, Seidler, 2011, Bock, 2005, Buch, Young, Contreras-Vidal, 2003, Fernández-Ruiz, Hall, Vergara, Díaz, 2000, Hegele, Heuer, 2008, Huang, Ahmed, 2014, Malone, Bastian, 2016, Nemanich, Earhart, 2015, Panouillères, Joundi, Brittain, Jenkinson, 2015, Roller, Cohen, Kimball, Bloomberg, 2002, Vandevoorde, Orban de Xivry, 2019, Wolpe, Ingram, Tsvetanov, Henson, Wolpert, Tyler, Brayne, Bullmore, Calder, Cusack, Dalgleish, Duncan, Matthews, Marslen-Wilson, Shafto, Campbell, Cheung, Davis, Geerligs, Kievit, McCarrey, Mustafa, Price, Samu, Taylor, Treder, van Belle, Williams, Bates, Emery, Erzinçlioglu, Gadie, Gerbase, Georgieva, Hanley, Parkin, Troy, Auer, Correia, Gao, Green, Henriques, Allen, Amery, Amunts, Barcroft, Castle, Dias, Dowrick, Fair, Fisher, Goulding, Grewal, Hale, Hilton, Johnson, Johnston, Kavanagh-Williamson, Kwasniewska, McMinn, Norman, Penrose, Roby, Rowland, Sargeant, Squire, Stevens, Stoddart, Stone, Thompson, Yazlik, Barnes, Dixon, Hillman, Mitchell, Villis, Rowe, 2020). Instead, our work reveals that a specific sub-domain of adaptation – long-term memory – is naturally enhanced in older adults and provides a neurochemical explanation of this phenomenon. Whether these findings are specific to reach adaptation, or prisms, or may also generalize to other effectors and forms of adaptation remains to be investigated. Regardless, they are relevant for translational research on ageing and stroke rehabilitation. Adaptation has been regarded as of limited value for rehabilitation because memory for what is learned decays so quickly (Kitago and Krakauer, 2013). Hence, much research effort (including by us) has been invested in developing neuroscience interventions to boost retention (Buch, Santarnecchi, Antal, Born, Celnik, Classen, Gerloff, Hallett, Hummel, Nitsche, Pascual-Leone, Paulus, Reis, Robertson, Rothwell, Sandrini, Schambra, Wassermann, Ziemann, Cohen, 2017, Galea, Mallia, Rothwell, Diedrichsen, 2015, Galea, Vazquez, Pasricha, Orban de Xivry, Celnik, 2010, O’Shea, Revol, Cousijn, Near, Petitet, Jacquin-Courtois, Johansen-Berg, Rode, Rossetti, 2017, Quattrocchi, Greenwood, Rothwell, Galea, Bestmann, 2017). Our finding that retention is naturally upregulated in (healthy) older age suggests such effort might be misplaced. If retention is already boosted naturally by ageing, then optimizing training regimens to exploit this for functional gain may instead prove a more profitable focus. This applies to health and disease. For example, postural imbalance, a cause of falls in older age, is associated with M1 GABA decline (Papegaaij, Taube, Hogenhout, Baudry, Hortobágyi, 2014, Swanson, Fling, 2018). Balance board training, used to counteract this, might be enhanced by incorporating an adaptation component, with the logic that the same GABA decline could promote retention of adapted training effects. Similarly, random as opposed to blocked practice impairs motor learning but boosts retention (Chalavi, Pauwels, Heise, Zivari Adab, Maes, Puts, Edden, Swinnen, 2018, Pauwels, Chalavi, Gooijers, Maes, Albouy, Sunaert, Swinnen, 2018). Leveraging this psychological insight, while capitalizing on naturally greater retention of adaptation in older age, may inspire the design of novel training regimes that better promote the maintenance of motor skills and thus functional independence through older age.

Is enhanced persistence likely to be beneficial in older adults? That will vary with context. Adaptation adjusts behaviour to counteract perturbations that impair performance, thus maintaining motor success. Once adapted, the optimal timescale for retention is the one that matches that of the perturbation (Kording et al., 2007). For long-lasting, slowly evolving perturbations, such as gradual muscle stiffening with increasing age, adapting to that and maintaining it over time would help to offset these deleterious effects. Conversely, in volatile environments that require agents to quickly learn and forget new visuomotor transformations (e.g. playing basketball on a windy day), slow forgetting would be maladaptive. Hence, whether reduced M1 inhibitory tone and stronger retention is adaptive or maladaptive depends on the context and the task. For instance, if prism lenses are never encountered again, then retaining a memory of the adapted state will yield no practical benefit. Stronger adaptation memory in older age is therefore best conceived of as a “paradoxical functional facilitation” (Kapur, 1996) – an isolated domain of upregulated function that may have benefits in some contexts, but which is a side effect of a deleterious process (age-related GABA loss) that primarily causes functional decline (Heise, Zimerman, Hoppe, Gerloff, Wegscheider, Hummel, 2013, Hermans, Leunissen, Pauwels, Cuypers, Peeters, Puts, Edden, Swinnen, 2018, King, Rumpf, Verbaanderd, Heise, Dolfen, Sunaert, Doyon, Classen, Mantini, Puts, Edden, Albouy, Swinnen, 2020, Papegaaij, Taube, Hogenhout, Baudry, Hortobágyi, 2014, Swanson, Fling, 2018).

## 5 Limitations

The main caveat of the present work is that only men were tested. This choice was informed by the fact that GABA levels change across the menstrual cycle in women and hence adaptation memory, E:I, and stimulation responsivity are also likely to fluctuate accordingly. Given hormonal changes across the lifespan, women’s M1 inhibitory tone may have a different or more variable age-related trajectory than men. Hence, to rule out these hormonal sources of variance, which would require much larger samples, we did not recruit women. In so doing, we follow a long tradition in biomedical research, the limitations and adverse consequences of which for women are significant (Perez, 2019). Of note, in our previous work, all three patient cases (by coincidence) were men (O’Shea et al., 2017). This may matter for the therapeutic effects observed. By excluding gender-related heterogeneity, we could identify an important mediator (M1 E:I) of variation in response to stimulation-induced functional plasticity, at least in men. Dissecting out intrinsic biological factors in this way helps to causally explain inter-individual differences and to dispel scepticism that this variability somehow renders brain stimulation (and tDCS in particular) suspect as a neuroscience tool (Horvath et al., 2014).

Another limitation of this study is that, due to practical reasons, the scanning protocol did not include a measure of the change in neurochemistry induced by M1 a-tDCS. The rationale for Experiment 2 and the basis for its interpretation (Fig. 6 & S4) rely on the premise that M1 a-tDCS directly increases E:I, at least in part via a decrease in GABAergic inhibition. The evidence for this comes from the tDCS-MRS literature in young adults (Bachtiar, Near, Johansen-Berg, Stagg, 2015, Barron, Vogels, Emir, Makin, O’Shea, Clare, Jbabdi, Dolan, Behrens, 2016, Kim, Stephenson, Morris, Jackson, 2014, Patel, Romanzetti, Pellicano, Nitsche, Reetz, Binkofski, 2019, Stagg, Best, Stephenson, O’Shea, Wylezinska, Kincses, Morris, Matthews, Johansen-Berg, 2009), and the same effect has been observed in older people (Antonenko et al., 2017) though inconsistently (King et al., 2020). In fact, the latter study found a positive relationship between age and the change in GABA concentration induced by M1 a-tDCS, such that older adults (above ∼65 years) no longer showed the GABA decrease seen in younger adults did but instead showed an increase. Similarly, another recent study reported a weakening of the excitatory effect of M1 a-tDCS on motor evoked potentials (MEPs) with older age (Ghasemian-Shirvan et al., 2020). These observations are all consistent with our interpretation of the results of Experiment 2. Future work should nevertheless confirm directly whether individuals who show the greatest increase in M1 E:I with a-tDCS exhibit the greatest increase in retention, while those who show no change or even a decrease in E:I (paradoxical, but see King et al., 2020) show a limited or reversed behavioural response to a-tDCS.

Finally, the present work suffers from limitations inherent to the MRS measurement technique. First, good signal-to-noise ratio was obtained by acquiring data from a large 2 × 2 × 2 cm^3^ MRS voxel centred on the region of interest. Due to the size of the voxel, adjacent regions of somatosensory cortex (S1) were also included in the measure of M1 metabolites. Although we cannot rule out the contribution of S1 to our results, M1 is likely to play a predominant role because of a convergence of studies implicating this region in the consolidation of adaptation memory (for review, see Panico et al., 2021). Second, it is well-established that the central nervous system includes at least two distinct forms of GABAergic inhibition: a tonic one involving (mostly extrasynaptic) metabotropic GABA_A_ receptors, and a phasic one involving (mostly intrasynaptic) ionotropic GABA_B_ receptors. Although resting MRS-GABA signal is thought to predominantly reflect the former (Stagg, Bestmann, Constantinescu, Moreno Moreno, Allman, Mekle, Woolrich, Near, Johansen-Berg, Rothwell, 2011, Stagg, Best, Stephenson, O’Shea, Wylezinska, Kincses, Morris, Matthews, Johansen-Berg, 2009), the latter may also explain some of the inter-individual variance in signal, and is known to play a role in adaptation memory (Johnstone et al., 2021). The extent to which tonic versus phasic GABAergic signalling contributes to our findings therefore remains an open question for future research. Third, when measuring the E:I ratio, the current study only considered the quantity of Glx and GABA neurotransmitters in the region of interest. However, there may be additional regulatory/modulatory mechanisms (e.g. influencing receptor concentration/effectiveness) that affect the net E:I in ways that are not captured by the MRS-derived metric used here. Such mechanisms might be better captured by MEP-derived physiological measures, which are known to bear little relationship to MRS measures (Cuypers, Hehl, van Aalst, Chalavi, Mikkelsen, Van Laere, Dupont, Mantini, Swinnen, 2021, Stagg, Bestmann, Constantinescu, Moreno Moreno, Allman, Mekle, Woolrich, Near, Johansen-Berg, Rothwell, 2011).

## 6 Conclusion

Our findings identified a domain of adaptation that is naturally up-regulated in older adults due to a natural decline in GABAergic inhibition: persistence of the after-effect. This finding may provide grounds for optimism about healthy motor ageing. The usual narrative is one of decline and loss. Maybe we cannot “*teach an old dog new tricks*”, but we can instead focus effort on adapting and retaining existing skills, promoted by natural neurochemical changes that may contribute to maintaining motor function for longer.

## Data and Code Availability

All analysis code and data related to this paper are available on the Open Science Framework (https://osf.io/stkv2/). A summary table of the key measures of both experiments is provided in *Supplementary Materials* to offer an easy way to track an individual participant’s data across the various parts of the study.

## CRediT authorship contribution statement

**Pierre Petitet:** Conceptualization, Investigation, Formal analysis, Data curation, Visualization, Writing – original draft. **Gershon Spitz:** Investigation, Formal analysis, Data curation, Visualization, Writing – original draft. **Uzay E. Emir:** Methodology, Software. **Heidi Johansen-Berg:** Resources, Writing – review & editing, Supervision, Funding acquisition. **Jacinta O’Shea:** Conceptualization, Resources, Writing – review & editing, Supervision, Funding acquisition, Project administration.

## Declaration of Competing Interest

The authors declare no competing interests.

## Appendix A Supplementary materials

Supplementary Data Supplementary Raw Research Data (Summary table of the most important behavioural and MRS measures of Experiments 1 & 2). This is open data under the CC BY license http://creativecommons.org/licenses/by/4.0/ Supplementary Data S1

Supplementary Information Supplementary Information. This is open data under the CC BY license http://creativecommons.org/licenses/by/4.0/ Supplementary Data S2
