# Supplementary material for: Age‐related decline in cortical inhibitory tone strengthens motor memory
Source: Neuroimage. 2021 Dec 15;245:118681. doi: 10.1016/j.neuroimage.2021.118681 (PMC8752967; doi:10.1016/j.neuroimage.2021.118681)
Supplement: Supplementary Information — This is open data under the CC BY license http://creativecommons.org/licenses/by/4.0/ [file mmc2.pdf]

SUPPLEMENTARY METHODS

MRS data quality filtering procedure

To treat noise in MRS data, ongoing methods development aims to develop procedures that systematically identify bad quality data in an objective way that does not depend on visual inspection of spectra by an expert. Over the years, the method of Cramér-Rao Lower Bounds (CRLB) has become the gold standard for determining concentration estimate uncertainty (Cavassila et al., 2001). The *CRLB* represents the lowest possible standard deviation of all unbiased concentration estimates obtained from fitting the model to the data. It is typically used in its *relative* form — labelled *%CRLB* — as a percentage of the estimated metabolite concentration. Its calculation for all estimated metabolites is part of the standard analysis pipeline of LCModel (Provencher, 1993, 2001). Many authors have used the *%CRLB* as a way to perform quality filtering by rejecting metabolite concentration estimates for which the *%CRLB* falls above a certain arbitrary threshold (usually between 20% and 50%), which is judged as an unacceptable level of uncertainty in the estimate (Kim et al., 2014; Barron et al., 2016; Stone et al., 2012; Rowland et al., 2012; Bachtiar et al., 2015).

Recently, however, some authors have warned against the usage of *%CRLB* for quality filtering because it could lead to wrong or missed statistical findings (Tisell et al., 2013; Kreis, 2016). For samples with large levels of noise, such as caused by bad quality MRS acquisition (e.g. too small voxel size, not enough averages, bad shimming) or bad quality spectrum fitting (e.g. inappropriate basis files), metabolite concentration estimates would be associated with high *%CRLB*, in a way that truly reflects high estimation uncertainty. In this scenario, it would be valid to mistrust the data based on a high *%CRLB*. However, because of the *relative* nature of the *%CRLB*, this metric also strongly depends on its denominator, i.e. the estimated metabolite concentration. Hence, for two samples acquired with equivalent levels of noise, the sample with a lower metabolite concentration will have a higher *%CRLB*. In that scenario, rejecting the dataset based on interpreting its high *%CRLB* as an indicator of high estimation uncertainty would be invalid.

This is relevant to the present study, in which we aimed to measure a reduction in GABA concentration with older age. The *%CRLB* cutoff criterion introduces a potential selection bias that could artificially bias the sample towards excluding participants with low GABA concentrations (and correspondingly high *%CRLB*). Therefore, to avoid this potential methodological confound we developed a data quality filtering approach that did not solely consider high *%CRLB* with respect to an arbitrary cutoff, but also considered the concentration estimate itself when deciding whether or not to reject datasets for quality control. Thus we aimed to better deal with the following scenarios: 1) datasets with a high *%CRLB* because of a low concentration estimate, rather than an excessive level of noise – such datasets should not be excluded; 2) Datasets with a low *%CRLB* simply because the concentration estimate is high might in fact be excessively high, given the metabolite concentration – such datasets should be excluded.

We therefore used the following method as an alternative to standard *%CRLB*-cutoff-based quality filtering. First, the following model was fitted to the "concentration estimate × *%CRLB*" relationship:

$$Expected\ \%CRLB_i = \frac{N_i}{C_i} \tag{1}$$

where N_i represents a group noise constant and C_i the concentration estimates for a metabolite i. Across the group, if this simple model can explain most of the variance in the observed relationship between concentration estimates and *%CRLB*, it means that the level of noise is relatively constant across all measurements. Any deviation from this model reflects an *unusual* level of noise compared to the other measurements. For each measurement, deviation from the model can be expressed as the Pearson residual as follows:

$$e_i = \frac{r}{\sqrt{MSE}} \tag{2}$$

where r_i is the raw residual (i.e. difference between the *%CRLB* and *expected %CRLB* for a certain measurement) and MSE is the mean squared error (i.e. mean deviation of all measurements from the model). The greater the Pearson residual for a given measurement, the noisier it is with respect to the rest of the data, irrespective of the concentration

estimate. Note that this method does not reject the lower tail of the distribution entirely and therefore does not induce a selection bias towards high concentration estimates. Datasets with a Pearson residual greater than 2 were considered excessively noisy and were excluded from statistical analysis.

SUPPLEMENTARY RESULTS

Effect of age during prism adaptation

In order to investigate the effect of age on pointing accuracy during adaptation, this between-subject variable (Age) was added to the two linear-mixed effect models (LMM) used to analyse CLP and OLP (Table S3 - models 1 & 2).

Age showed no association to OLP during PA (main effect and interactions: all $p > 0.10$). By contrast, as reported previously in the literature (Fernández-Ruiz et al., 2000; Buch et al., 2003; Bock, 2005; Anguera et al., 2011; Huang and Ahmed, 2014; Panouillères et al., 2015; Vandevoorde and Orban de Xivry, 2019), older age was associated with a slower rate of error correction on CLP blocks (interaction Age × Trial: $t_{(60.6)} = 2.59, p = 0.012$, $\eta_p^2 = 0.10, 95\%CI = [0.00, 0.20]$). This result was unchanged when controlling for movement duration of individual CLP trials.

To investigate whether the age-related increase in M1 E:I (Fig. 2) was responsible for this decline in error correction rate, Age was replaced by M1 E:I in the previous LMM (controlling for CLP movement duration). This did not yield any significant interaction between M1 E:I and Trial ($t_{(25.08)} = -0.13, p = 0.90$, $\eta_p^2 = 0.00, 95\%CI = [0.00, 0.10]$), suggesting that the age-related impairment of error correction on CLP was not related to M1 neurochemistry. Similarly, M1 E:I did not show any significant relationship to the development of an AE during PA (main effect and interactions: all $p > 0.10$).

Older age is not associated with a failure to de-adapt

In Experiment 1, older age was associated with a larger AE at 24-hours but not at 10-minutes after PA (Fig. 1). One possible explanation for this pattern of relationship is that older age leads to a memory trace that is more robust to washout. According to this hypothesis, active de-adaptation (i.e. visually guided movements performed without prism glasses) may more strongly wash out the AE in younger than older individuals, which would explain why the relationship between age and retention becomes more apparent at a later time point. In order to test this hypothesis, the protocol of Experiment 2 included a short de-adaptation phase immediately after the 10-minute retention time point. During this phase, participants alternated between three blocks of closed-loop pointing (10-10-20 trials) and three blocks of open-loop pointing (15-15-15 trials). A linear mixed-effect model was built for each pointing type to investigate the effect of age on within- and across- block dynamics of endpoint errors in the sham condition.

On the first CLP trial of washout, participants made a large leftward error (mean −5.28°, s.e.m.: 2.87°, one-sample t-test compared to zero: $t_{(24)} = -9.19$, $p < 0.001$, Cohen's d = -1.84, $95\%CI = [-2.53, -1.21]$), indicative of the presence of an AE on this type of pointing as well. Because they received visual feedback of their reach endpoints, participants were able to reduce the magnitude of this error within and across the three washout blocks (main effect of Trial within Block: $t_{(28.18)} = 9.73, p < 0.001$, $\eta_p^2 = 0.77$, $95\%CI = [0.60, 0.85]$; main effect of Block: $t_{(28.22)} = 9.28, p < 0.001$, $\eta_p^2 = 0.75, [95\%CI : 0.57, 0.84]$). The slope of this active de-adaptation flattened as errors converged towards zero (interaction Trial × Block: $t_{(43.28)} = -9.32$, $p < 0.001$, $\eta_p^2 = 0.67$, $[95\%CI = [0.50, 0.77]]$. Crucially, the LMM showed no significant main effect of age or interaction of age with the other effects during active de-adaptation (all $p > 0.05$). In other words, we found no evidence that older age was associated with a failure to de-adapt once prism lenses were removed.

Although older age was not associated with a deficit in de-adaptation *per se* on CLP, we found that it was linked to a stronger resistance of the AE to washout on OLP. Overall, open-loop pointing behaviour was characterised by spontaneous recovery (also called "rebound") during washout, i.e. a gradual reappearance of a (negative) leftward AE within each interleaved AE block (main effect of Trial within Block: $t_{(25)} = -6.61, p < 0.001$, $\eta_p^2 = 0.64$, $95\%CI = [0.38, 0.77]$). This is a well-known phenomenon in the adaptation literature thought to result from the competition

between learning and memory processes unfolding over different timescales (Smith et al., 2006; Kording et al., 2007). This spontaneous recovery was found to be relatively preserved across the three OLP blocks (interaction Trial × Block: $t_{(70.06)} = 0.33$, $p = 0.74$, $\eta_p^2 = 0.00$, $95\%CI = [0.00, 0.06]$) such that, on average, the AE remained constant throughout washout (main effect of Block: $t_{(25)} = 0.12, p = 0.90$, $\eta_p^2 = 0.00$, $95\%CI = [0.00, 0.10]$). Age was found to interact with the way in which spontaneous recovery evolved across successive blocks (interaction Age *times* Trial × Block: $t_{(70.06)} = -2.19$, $p = 0.032$, $\eta_p^2 = 0.06$, $95\%CI = [0.00, 0.20]$). That is, in older individuals, the slope of spontaneous recovery tended to become steeper over successive OLP blocks, instead of the decrease in the slope observed in younger people (see Fig. S5 for illustration purposes). This indicates stronger re-activation of adaptation memory during washout in older participants (Fig. S5).

In order to check that this finding was not merely caused by older people having a greater natural tendency to shift leftward during open-loop pointing in general, the effect of age was also analysed at baseline, before any adaptation took place. At baseline, older age was not systematically associated with more left-shifted pointing errors during OLP (main effect of Age: $t_{(725)} = 0.00$, $p = 0.99$, $\eta_p^2 = 0$, $95\%CI = [0, 0]$) nor with a tendency to shift leftward over trials (interaction Age *times* Trial: $t_{(25)} = 0.97$, $p = 0.34$, $\eta_p^2 = 0.04$, $[95\%CI = [0, 0.26]$). The same analysis of the baseline OLP block of Experiment 1 confirmed the same absence of effect of age ($p > 0.05$).

Neurochemistry significantly moderates how stimulation changes retention but age does not

Given the inter-relationships between age, neurochemistry and retention we observed in Experiment 1 (Figs. 2-4),we also tested whether age (and not just M1 E:I) moderated how stimulation changed memory (in Experiment 2), and hence could serve as a (cheaper and easier to measure) surrogate of M1 E:I to predict stimulation response.

Age did not moderate the effect of stimulation on retention (age × a-tDCS: $t_{(1419)} = 0.79$, $p = 0.43$, two-tail). There was no association between age and the effect of a-tDCS on long-term retention (Fig. S3). This was the case when the full sample of all participants in Experiment 2 was analysed ($n = 25$; $r_{(23)} = -0.12$, $p = 0.56$). It was also true of the reduced sample of participants for whom we also had MRS data ($n = 16$; $r_{(14)} = 0.19$, $p = 0.47$). To directly compare the relative impacts of age versus M1 E:I on the behavioural effect of a-tDCS, we used Fisher's r-to-z transform to contrast their Pearson correlation coefficients (i.e. relationships in Fig. 6a versus Fig S3). The relationship between the stimulation effect and M1 E:I was significantly greater than that between the stimulation effect and age for the full dataset ($t_{(14)} = -2.53$, $p = 0.02$). For the reduced sample, correlations did not differ significantly, presumably reflecting lower statistical power ($t_{(14)} = -0.97$, $p = 0.35$). We conclude that mere chronological age does not predict the effect of stimulation on retention, by contrast with M1 E:I.

SUPPLEMENTARY FIGURE & TABLES

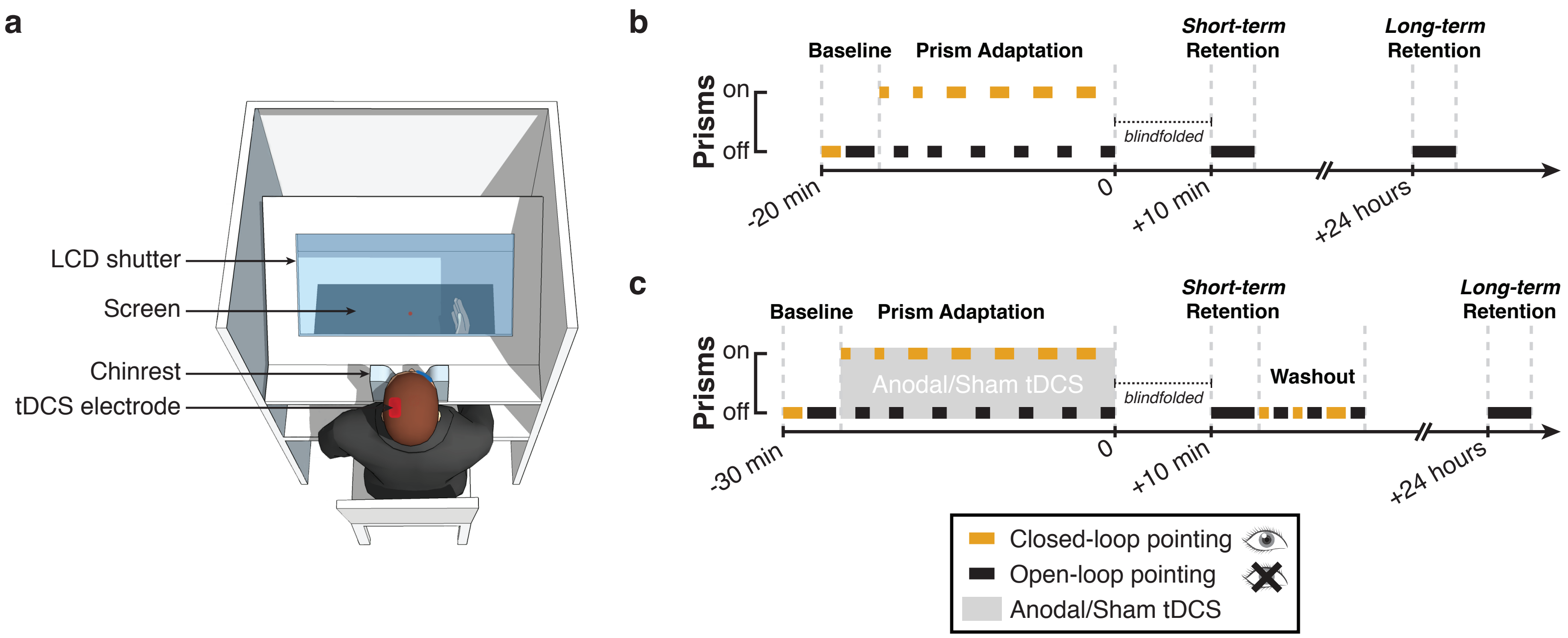

Figure S1: **Prism adaptation protocol. a. Experimental setup.** For both Experiment 1 and 2, participants sat in a chinrest viewing a horizontal 32-inch touchscreen through a liquid crystal shutter. The touchscreen was used to present visual targets and record reach endpoints. The liquid crystal display shutter was used to control visual feedback by turning opaque during reaching movements to conceal endpoint performance. **b. Procedure for Experiment 1.** Baseline accuracy was measured without prisms during blocks of closed-loop (continuous visual feedback) and open-loop (no visual feedback) pointing. During adaptation, participants alternated between blocks of prism exposure (closed-loop, glasses on) and after-effect measurement (open-loop, prisms off). Retention of the after-effect was measured 10 minutes and 24 hours post-adaptation. **c. Procedure for Experiment 2.** The procedure for Experiment 2 was the same as Experiment 1, except that left M1 anodal tDCS (real/sham) was applied throughout adaptation (grey shading). Short-term retention was followed by washout, during which participants observed and corrected their leftward errors (closed-loop pointing blocks, no prisms), interleaved with open-loop measures to confirm after-effect decay back to baseline.

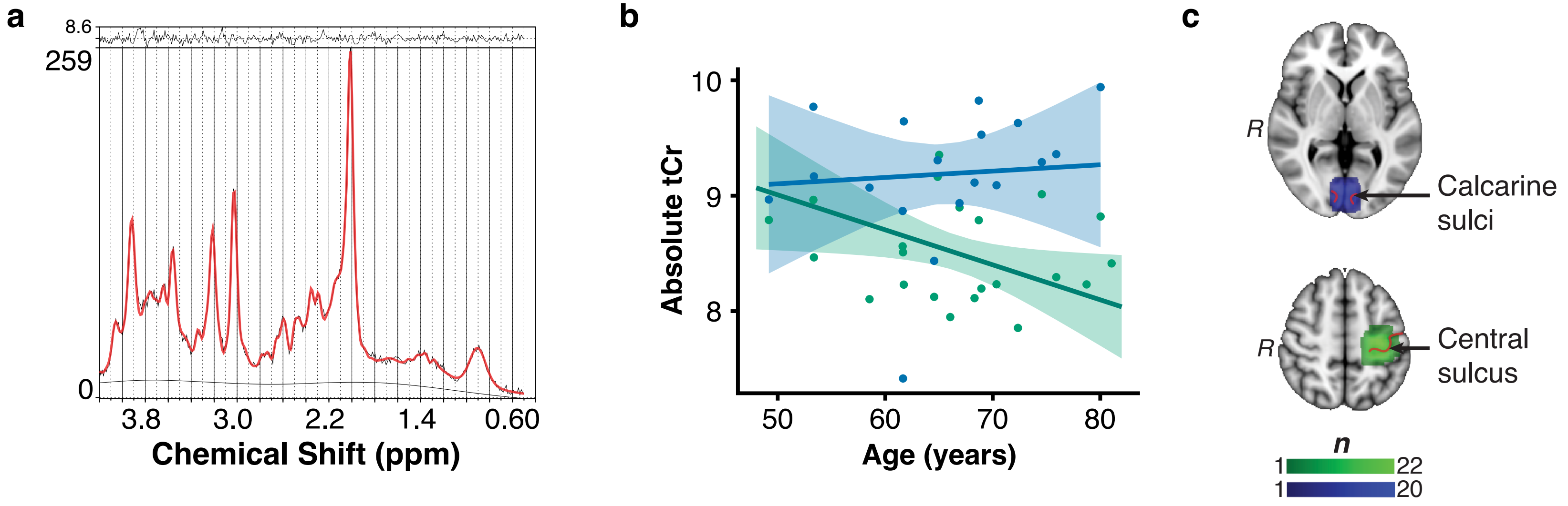

Figure S2: **Magnetic resonance spectroscopy data quality.** This figure shows the quality of MRS data collected in Experiment 1. **a.** Example raw MRS spectrum and LCModel fit from one participant. The fitted LCModel (in red) is plotted overlaid on the raw data (in black). The difference between the data and model (residuals) is shown at the top and the baseline is shown at the bottom. **b.** This panel presents the association between age and total Creatine (tCr), controlling for the fraction of WM and GM, in the M1 voxel (in green) and the V1 voxel (in blue). Shading indicates 95% confidence intervals. This panel shows that the tCr estimate was negatively correlated with age in M1, but not in V1. Because of this relationship, we use absolute concentrations of GABA and Glx throughout the paper, rather than using tCr for internal referencing. **c.** Magnetic resonance spectroscopy voxels group overlap map. The M1 voxel was centred on the left central sulcus in 22 participants (in green, MNI coordinate z = 52). The control V1 voxel was centred on the bilateral calcarine sulcus in 20 participants (in blue, MNI coordinate z = 2). Colour bar represents the degree of overlap. All images are displayed in radiological convention (i.e. left side of the image corresponds to the right side of the brain).

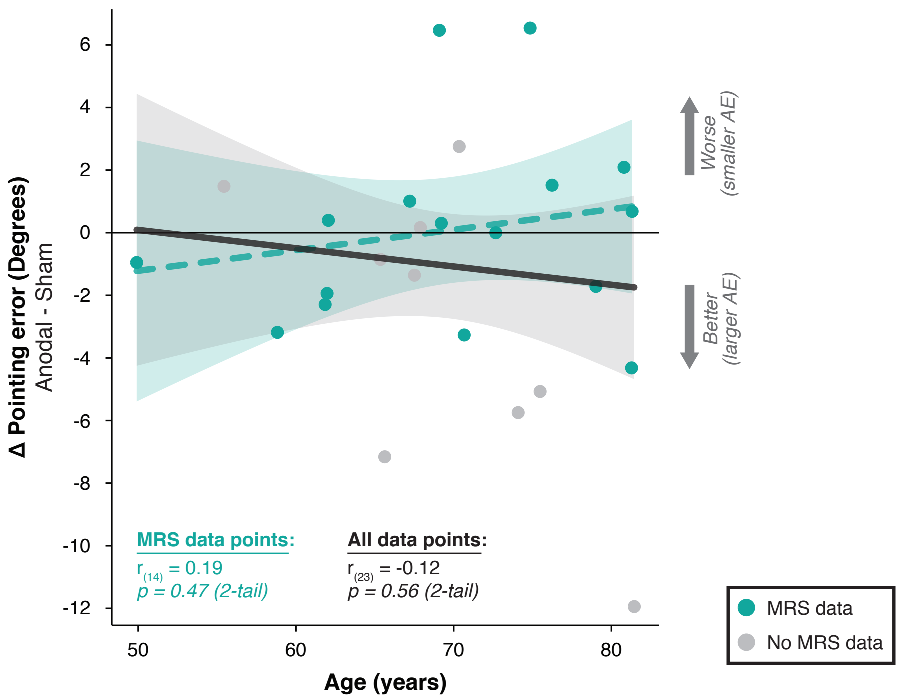

Figure S3: **The behavioural effect of a-tDCS on long-term retention is not moderated by age.** Age is plotted against the stimulation effect (anodal - sham difference in normalised pointing error at 24-hour retention). On the y-axis, negative values indicate greater retention with anodal tDCS compared to sham. Positive values indicate the opposite. The linear regression line is plotted for both the reduced sample of participants who had both MRS and retention data (n = 16, blue data points and line) as well as the full sample – including those participants who had missing MRS data (n = 25, black line). Irrespective of the sample considered, there was no association between age and the effect of a-tDCS on long-term retention (both $p > 0.05$).

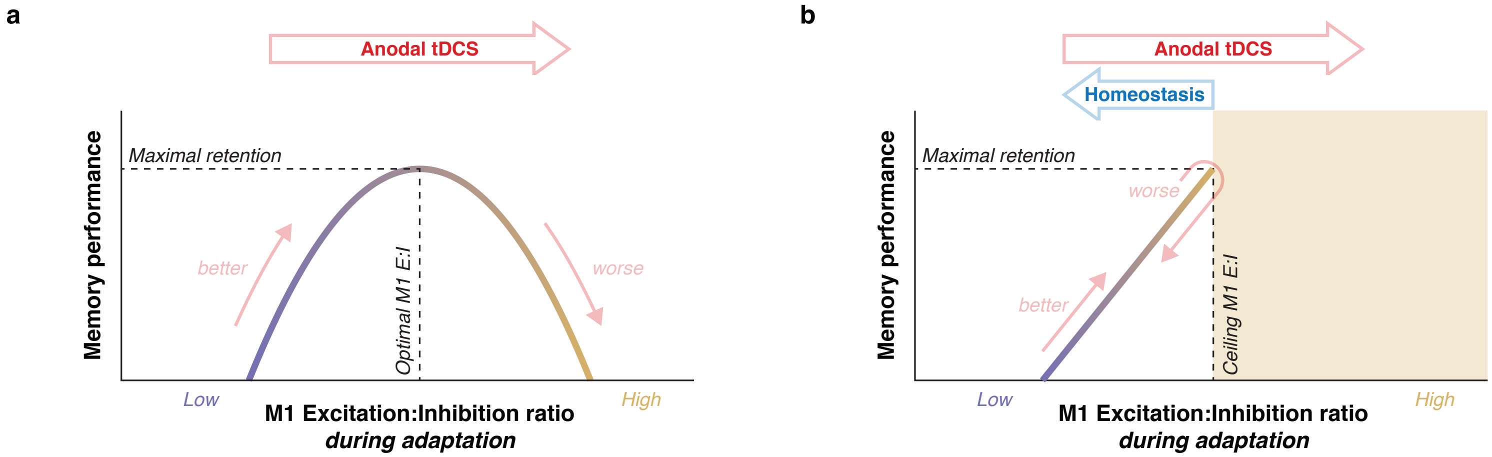

Figure S4: **Alternative account of how stimulation impairs adaptation memory in individuals with high M1 E:I. a** For comparison purposes, the model proposed in Fig. 6b is reproduced here. **b.** The schematic offers an alternative mechanistic interpretation of the data presented in Fig. 6a. This model assumes that in a healthy brain there is a ceiling on cortical excitation which, when exceeded, triggers homeostatic mechanisms that reduce E:I, to bring it back within physiological range. In individuals with naturally high M1 E:I (near ceiling), homeostasis could overshoot, leading to an overall decrease in E:I. This mechanism could explain why stimulation impairs retention in those with high baseline M1 E:I, without requiring a reversal in the sign of the relationship between M1 E:I and retention.

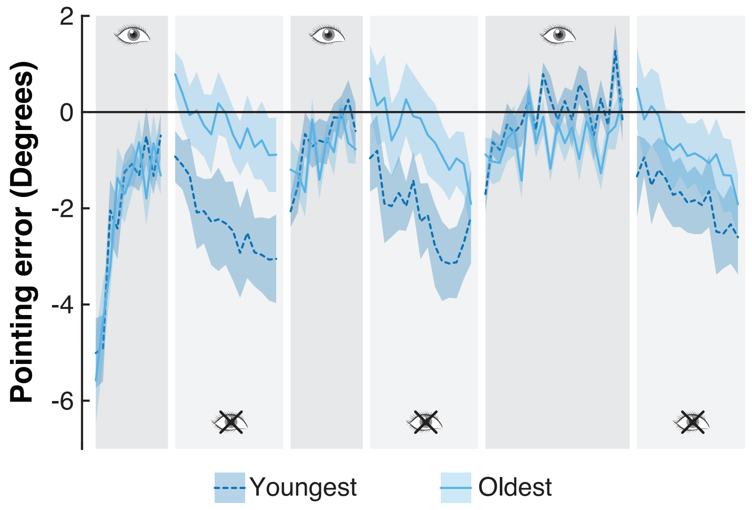

Figure S5: **Active de-adaptation data in Experiment 2 – sham tDCS condition.** Group mean pointing errors expressed as change from baseline accuracy ($y = 0$). Positive y-axis values are rightward errors (i.e. in the direction of the prismatic shift), negative leftward. Error bands indicate s.e.m. During closed-loop pointing (dark grey background), endpoint errors were initially deviated in the direction opposite to the prismatic shift, due to the adaptation that took place before. Visual feedback enabled participants to correct their initially leftward pointing errors. After-effects were measured in intervening blocks without visual feedback (light grey background). A leftward after-effect spontaneously re-appeared in each of these three blocks. The data are split by age (median split; $n = 13$ youngest participants in dotted dark blue line, $n = 12$ oldest participants in full light blue line).

		**Experiment 1**		**Experiment 2**
**Participant**	**Age**	*PA session*	*MRS*	*PA+tDCS sessions*
**1**	81.1	✓	(✓)	✓
**2**	78.7	✓	(✓)	✓
**3**	80.0	✓	✓	✓
**4**	75.9	✓	✓	✓
**5**	74.6	✓	✓	✓
**6**	72.3	✓	✓	✓
**7**	68.7	✓	✓	✓
**8**	66.9	✓	✓	✓
**9**	64.6	✓	✓	
**10**	53.3	✓	✓	
**11**	68.9	✓	✓	✓
**12**	80.7	✓		✓
**13**	70.4	✓	✓	✓
**14**	66.0	✓	✓	
**15**	61.7	✓	✓	✓
**16**	68.3	✓	✓	
**17**	58.5	✓	✓	✓
**18**	53.4	✓		
**19**	64.9	✓	✓	
**20**	61.7	✓	✓	✓
**21**	49.2	✓	✓	
**22**	61.6	✓	✓	✓
**23**	65.0	✓	✓	✓
**24**	65.6	✓	✓	✓
**25**	75.4	✓		✓
**26**	74.1	✓		✓
**27**	67.8	✓		✓
**28**	65.2	✓		✓
**29**	55.4	✓		✓
**30**	67.4	✓		✓
**31**	70.3	✓		✓
**32**	70.9	✓		✓

Table S1: **Participant demographics and inclusion details.** Experiment 1 consisted of a behavioural experiment (prism adaptation, PA; with retention probe the next day) and a MR Spectroscopy scan. Experiment 2 consisted of two PA sessions plus retention probes 24 hours later, separated by one week. Tick marks indicate which measures were obtained for which participants. In the MRS column, parentheses indicate incomplete data due to technical difficulties (missing control voxel).

Participant	Voxel	GM	WM	CSF	FWHM	SNR	CRLB (GABA)	CRLB (Glx)
1	M1	19.79	56.58	23.63	0.036	35	24	6
2	M1	19.84	66.32	13.84	0.048	31	21	5
3	M1	19.50	62.99	17.51	0.024	35	34	7
4	M1	20.68	56.34	22.99	0.048	39	23	4
5	M1	24.10	46.12	29.79	0.036	31	23	4
6	M1	29.63	55.30	15.07	0.036	35	24	6
7	M1	26.99	58.13	14.88	0.036	41	31	5
8	M1	25.85	58.52	15.63	0.048	36	19	4
9	M1	24.89	63.06	12.05	0.048	37	24	4
10	M1	30.13	60.06	9.81	0.036	35	25	6
11	M1	27.02	64.41	8.57	0.036	43	26	5
13	M1	23.74	61.53	14.73	0.048	34	22	5
14	M1	28.64	53.80	17.56	0.036	42	19	5
15	M1	24.63	61.86	13.51	0.036	38	21	5
16	M1	25.15	59.97	14.88	0.048	32	29	6
17	M1	24.25	69.98	5.77	0.048	35	23	6
19	M1	29.71	54.34	15.96	0.036	36	20	6
20	M1	23.26	57.15	19.59	0.036	33	20	6
21	M1	20.37	68.78	10.85	0.036	36	27	7
22	M1	30.82	51.41	17.77	0.036	35	20	5
23	M1	29.36	55.34	15.30	0.048	39	26	5
24	M1	22.72	59.81	17.47	0.048	39	18	5
1	V1	*Not acquired*						
2	V1	*Not acquired*						
3	V1	48.51	29.48	22.01	0.036	39	25	5
4	V1	52.00	20.39	27.61	0.048	38	30	4
5	V1	47.26	30.44	22.22	0.036	44	22	4
6	V1	50.06	24.07	25.87	0.048	38	22	4
7	V1	47.90	30.28	21.80	0.036	41	19	5
8	V1	44.54	37.33	18.13	0.048	43	21	5
9	V1	46.64	34.90	18.47	0.036	44	26	4
10	V1	53.85	28.87	17.27	0.048	39	21	4
11	V1	54.21	31.50	14.30	0.048	46	18	4
13	V1	43.67	39.36	16.98	0.036	47	21	4
14	V1	50.81	32.97	16.22	0.048	17	120*	14*
15	V1	51.95	34.06	13.99	0.048	26	35*	8
16	V1	40.91	33.66	25.43	0.048	34	26	5
17	V1	56.66	28.67	14.67	0.036	41	17	4
19	V1	55.35	29.95	14.70	0.059	32	30*	6
20	V1	47.20	36.26	16.54	0.036	40	23	5
21	V1	45.02	31.64	23.34	0.048	40	19	4
22	V1	59.21	27.61	13.18	0.036	45	18	5
23	V1	46.02	37.00	16.84	0.036	47	19	4
24	V1	51.37	33.83	14.61	0.048	49	19*	4*

Table S2: **MRS data quality metrics.** GM: Fraction of Grey Matter; WM: Fraction of White Matter; CSF: Fraction of Cerebrospinal Fluid; SNR: Signal/Noise Ratio; CRLB: Cramer–Rao Bounds; FWHM: Full-Width Half Maximum; *: datasets that failed quality filtering.

	Dependent variable:			
	Normalised angular error			
	Closed-loop pointing	Open-loop pointing	10-min retention	24-hour retention
	(1)	(2)	(3)	(4)
Intercept	1.06** (0.84, 1.27)	−6.66** (−7.43, −5.89)	−4.61** (−5.40, −3.81)	−1.30** (−2.23, −0.37)
Trial	−0.08** (−0.10, −0.07)	0.14** (0.11, 0.17)	0.01 (−0.01, 0.03)	0.002 (−0.02, 0.02)
Block	−0.42** (−0.51, −0.33)	−0.36** (−0.55, −0.18)		
Trial:Block	0.05** (0.04, 0.06)	−0.02** (−0.03, −0.01)		
Observations	3,200	2,880	1,440	1,440
Bayesian Inf. Crit.	13,771.00	10,153.74	5,183.54	4,949.39

Table S3: **Experiment 1: Prism adaptation behaviour.** All LMMs analysed the normalised pointing error as the dependent variable (i.e. trial endpoint errors *minus* mean baseline error). Model (1) assesses the reduction of CLP errors throughout prism exposure (blocks E1-6), while model (2) captures the development of an after-effect on OLP trials (blocks AE1-6). Models (3) and (4) assess the persistence (intercept) and stability (main effect of Trial) of the after-effect (OLP) at the 10-minutes and 24-hours retention intervals. $^{*}p<0.05$; $^{**}p<0.01$ (all two-tailed).

	Dependent variable:					
	Normalised angular error					
	End of PA	10-min retention	24-hour retention	24-hour retention [c:AE6]	24-hour retention [controlling:10-min]	24-hour retention [c:mvtDuration]
	(1)	(2)	(3)	(4)	(5)	(6)
Intercept	−7.36** (−8.46, −6.26)	−4.61** (−5.39, −3.82)	−1.30** (−2.16, −0.44)	−1.30** (−2.16, −0.44)	−1.30** (−2.16, −0.44)	−0.07 (−3.36, 3.21)
Trial	0.10** (0.07, 0.14)	0.01 (−0.01, 0.03)	0.002 (−0.02, 0.02)	0.002 (−0.02, 0.02)	0.002 (−0.02, 0.02)	0.002 (−0.02, 0.02)
Age	0.03 (−0.11, 0.17)	0.05 (−0.05, 0.15)	−0.12* (−0.23, −0.02)	−0.13* (−0.24, −0.02)	−0.13* (−0.24, −0.02)	−0.12* (−0.23, −0.01)
Age:Trial	0.0005 (−0.004, 0.01)	0.002 (−0.0003, 0.004)	0.001 (−0.002, 0.004)	0.001 (−0.002, 0.004)	0.001 (−0.002, 0.004)	0.001 (−0.002, 0.004)
mean AE (end of PA)				0.11 (−0.14, 0.37)		
mean AE (10-min)					0.03 (−0.33, 0.40)	
Movement duration						−0.003 (−0.01, 0.004)
Observations	480	1,440	1,440	1,440	1,440	1,440
Bayesian Inf. Crit.	1,730.39	5,194.85	4,957.99	4,964.52	4,965.23	4,964.71

Table S4: **Experiment 1: Older participants show stronger long-term retention** The LMMs reported in this table examine the relationship between age and prism adaptation memory. The normalised pointing error was the dependent variable (i.e. trial endpoint errors *minus* mean baseline error). Models (1), (2) and (3) examine the relationship between age and prism after-effect at the end of adaptation (block AE6), and at the 10-minutes and 24-hour retention time points, respectively. Only 24-hour retention was related to age, such that older participants showed a larger (more negative) after-effect (AE). The next three models assess the robustness of this result when controlling for the average AE at the end of adaptation (model 4), the average AE at the 10-minutes retention interval (model 5), and the average movement duration on CLP trials during prism exposure (model 6). The relationship between age and long-term adaptation memory survived controlling for all three factors, confirming that it was not an artefact of older participants adapting to a greater extent on the first day or pointing more slowly. *$p<0.05$; **$p<0.01$ (all two-tailed).

	Dependent variable:					
	M1 E:I	M1 GABA	M1 Glx	V1 E:I	V1 GABA	V1 Glx
	(1)	(2)	(3)	(4)	(5)	(6)
Intercept	−0.00 (−0.41, 0.41)	0.00 (−0.14, 0.14)	0.00 (−0.39, 0.39)	−0.22 (−0.73, 0.30)	0.08 (−0.12, 0.27)	−0.34* (−0.63, −0.05)
Age	0.08 (0.005, 0.15)	−0.03* (−0.06, −0.01)	−0.03 (−0.11, 0.05)	0.05 (−0.02, 0.12)	−0.02 (−0.04, 0.004)	0.005 (−0.04, 0.05)
Glx		0.04 (−0.13, 0.21)			0.28 (0.003, 0.56)	
GABA			0.31 (−0.98, 1.61)			0.93 (0.01, 1.85)
GM	0.15 (−0.04, 0.34)	−0.07 (−0.14, −0.0003)	−0.07 (−0.27, 0.13)	−0.10 (−0.24, 0.05)	0.05 (−0.003, 0.10)	−0.12* (−0.20, −0.03)
WM	0.04 (−0.05, 0.13)	−0.03 (−0.07, 0.01)	−0.12* (−0.22, −0.03)	−0.11 (−0.24, 0.02)	0.06 (−0.01, 0.14)	−0.22** (−0.29, −0.15)
Observations	22	22	22	16	16	16
Adjusted R^2	0.06	0.20	0.25	0.40	0.48	0.77

Table S5: **Experiment 1: Older participants have a higher excitation:inhibition ratio in sensorimotor cortex.** The linear regressions reported in this table examine the relationship between age and metabolite concentration within the motor (labelled "M1") and occipital (labelled "V1") cortex voxels. All models controlled for the fraction of grey and white matter within the MRS voxel, and included the MRS measure as the dependent variable. Model (1) shows the predicted significant positive relationship between age and E:I ratio (Glx:GABA). Models (2) and (3) decompose this relationship into its GABA and Glx constituents respectively. They highlight that the age-related increase in E:I was mainly due to a loss of GABA-ergic inhibition. The final three models show a qualitatively similar, though not significant, pattern within the bilateral occipital cortex. *$p<0.05$; **$p<0.01$ (all two-tailed).

	Dependent variable:							
	Normalised angular error							
	M1 E:I	M1 GABA and Glx	M1 E:I [c:mvtDuration]	M1 GABA and Glx [c:mvtDuration]	V1 E:I	V1 GABA and Glx	V1 E:I [c:mvtDuration]	V1 GABA and Glx [c:mvtDuration]
	(1)	(2)	(3)	(4)	(5)	(6)	(7)	(8)
Intercept	−0.79* (−1.53, −0.05)	−0.79* (−1.56, −0.03)	2.73 (−1.41, 6.87)	1.51 (−2.86, 5.87)	−0.44 (−2.07, 1.20)	0.08 (−1.75, 1.91)	10.95** (4.23, 17.67)	11.12** (4.14, 18.11)
Trial	0.01 (−0.03, 0.04)	0.01 (−0.03, 0.04)	0.01 (−0.03, 0.04)	0.01 (−0.03, 0.04)	−0.001 (−0.04, 0.04)	−0.003 (−0.04, 0.04)	−0.001 (−0.04, 0.04)	−0.003 (−0.04, 0.04)
EI	−2.07** (−2.82, −1.32)		−2.07** (−2.77, −1.37)		0.21 (−1.41, 1.82)		1.02 (−0.35, 2.39)	
GABA		5.59** (3.42, 7.76)		5.58** (3.49, 7.67)		−1.51 (−6.62, 3.60)		−3.32 (−7.63, 0.99)
Glx		0.003 (−0.87, 0.88)		−0.15 (−1.04, 0.74)		1.70 (−1.24, 4.63)		0.48 (−1.97, 2.94)
GM	0.24* (0.03, 0.45)	0.29** (0.09, 0.50)	0.15 (−0.07, 0.37)	0.23* (0.01, 0.45)	0.28 (−0.11, 0.67)	0.44 (−0.05, 0.94)	0.20 (−0.10, 0.50)	0.23 (−0.17, 0.64)
WM	−0.01 (−0.15, 0.12)	0.11 (−0.05, 0.27)	−0.03 (−0.16, 0.10)	0.08 (−0.08, 0.24)	0.30 (−0.08, 0.69)	0.65 (−0.06, 1.36)	0.18 (−0.12, 0.48)	0.18 (−0.45, 0.80)
Trial:EI	−0.01 (−0.04, 0.02)		−0.01 (−0.04, 0.02)		0.01 (−0.02, 0.05)		0.01 (−0.02, 0.05)	
Trial:GABA		0.01 (−0.08, 0.10)		0.01 (−0.08, 0.10)		−0.03 (−0.13, 0.08)		−0.03 (−0.13, 0.08)
Trial:Glx		−0.004 (−0.03, 0.03)		−0.004 (−0.03, 0.03)		0.01 (−0.03, 0.05)		0.01 (−0.03, 0.05)
Movement duration			−0.01 (−0.02, 0.001)	−0.01 (−0.02, 0.01)			−0.03** (−0.05, −0.01)	−0.03** (−0.05, −0.01)
Observations	990	990	990	990	720	720	720	720
Bayesian Inf. Crit.	3,359.62	3,371.64	3,363.89	3,377.51	2,479.94	2,491.97	2,477.90	2,490.77

Table S6: **Experiment 1: Higher sensorimotor cortex excitation:inhibition ratio is associated with greater 24-hour retention**. The LMMs reported in this table examine the relationship between M1 and V1 neurochemistry and the magnitude of the AE at 24-hours. All models controlled for the fraction of grey and white matter within the MRS voxel. Model (1) shows that individuals with higher M1 E:I had a larger (more negative) AE at 24-hours. Model (2) decomposes this relationship into its GABA and Glx constituents respectively, highlighting that GABA but not Glx drives the previous relationship. Models (3) and (4) show that these findings were robust to controlling for the average movement duration on CLP trials during prism exposure. Finally, models (5) to (8) reproduce the same set of analyses using MRS data from the anatomical control voxel (V1). No relationship between neurochemistry and long-term adaptation memory was observed in the V1 voxel. $^{*}p<0.05$; $^{**}p<0.01$ (all two-tailed).

	Dependent variable:					
	M_1 (M1 E:I)	M_2 (M1 GABA)	M_3 (M1 Glx)	Y (AE24hrs)		
	(1)	(2)	(3)	(4)	(5)	(6)
X (age)	0.66 (0.31)	-0.78^{*} (0.28)	−0.31 (0.27)	-0.76^{*} (0.29)	−0.35 (0.24)	−0.28 (0.28)
M_1 (M1 E:I)					-0.63^{**} (0.16)	
M_2 (M1 GABA)						0.65^{**} (0.20)
M_3 (M1 Glx)						−0.06 (0.20)
C_1 (GM)	0.54 (0.35)	-0.68^{*} (0.31)	−0.31 (0.30)	−0.40 (0.32)	−0.07 (0.25)	0.02 (0.29)
C_2 (WM)	0.20 (0.26)	-0.53^{*} (0.23)	-0.70^{**} (0.22)	−0.32 (0.23)	−0.19 (0.18)	−0.01 (0.25)
Observations	22	22	22	22	22	22
Adjusted R^2	0.06	0.24	0.28	0.22	0.56	0.48
F Statistic	1.46 (df = 3; 18)	3.16 (df = 3; 18)	3.72^{*} (df = 3; 18)	2.99 (df = 3; 18)	7.81^{**} (df = 4; 17)	4.88^{**} (df = 5; 16)

Table S7: **Experiment 1: Motor cortical GABA mediates the relationship between age and long-term adaptation memory.** Model (1) shows a near-significant relationship between age and motor cortical E:I ($p = 0.051$, two-tailed; $p = 0.025$, one-tailed). Models (2) and (3) show that this relationship is driven by GABA ($p = 0.013$) and not Glx ($p = 0.27$). Model (4) shows that older age is associated with greater 24-hour retention ($p = 0.02$). Crucially, model (5) demonstrates that the association between age and 24-hour retention is no longer significant when accounting for M1 E:I. Further, model (6) shows that the mediation is specifically driven by GABA ($p = 0.004$) and not Glx ($p = 0.78$). Overall, these regression models provide support in favour of M1 GABA mediating the relationship between age and long-term retention, which was subsequently assessed formally. The mediation analysis indicates a significant effect of M1 GABA ($ab_1 = -0.50$, $95\%CI : [-1.36, -0.14]$, $p = 0.01$) but not M1 Glx ($ab_2 = 0.02$, $95\%CI : [-0.09, 0.31]$, $p = 0.73$; see Fig. 4). *p<0.05; **p<0.01 (all two-tailed).

	Dependent variable:					
	M_1 (M1 E:I)	M_2 (M1 GABA)	M_3 (M1 Glx)	Y (AE24hrs)		
	(1)	(2)	(3)	(4)	(5)	(6)
X (age)	0.66 (0.31)	−0.78* (0.28)	−0.31 (0.27)	−0.68* (0.29)	−0.34 (0.24)	−0.28 (0.29)
M_1 (M1 E:I)					−0.60** (0.17)	
M_2 (M1 GABA)						0.66* (0.23)
M_3 (M1 Glx)						−0.05 (0.21)
C_1 (GM)	0.54 (0.35)	−0.68* (0.31)	−0.31 (0.30)	−0.31 (0.32)	−0.04 (0.26)	0.02 (0.30)
C_2 (WM)	0.20 (0.26)	−0.53* (0.23)	−0.70** (0.22)	−0.27 (0.23)	−0.18 (0.18)	−0.01 (0.27)
C_3 (Mvt duration)				−0.23 (0.20)	−0.09 (0.16)	0.02 (0.20)
Observations	22	22	22	22	22	22
Adjusted R^2	0.06	0.24	0.28	0.24	0.55	0.45
F Statistic	1.46 (df = 3; 18)	3.16 (df = 3; 18)	3.72* (df = 3; 18)	2.63 (df = 4; 17)	6.08** (df = 5; 16)	3.81* (df = 6; 15)

Table S8: **Experiment 1: Mediation analysis controlling for average CLP duration.** This table presents the results of the mediation analysis, controlling for the average reaching movement duration on CLP trials during prism exposure. Overall, this table indicates that the results presented in Table S7 are unchanged: M1 GABA, but not Glx, mediates the relationship between age and 24-hour retention. *p<0.05; **p<0.01 (all two-tailed).

	Dependent variable:				
	Normalised angular error				
	a-tDCS	a-tDCS × M1 E:I	a-tDCS × M1 (GABA,Glx)	a-tDCS × V1 E:I	a-tDCS × V1 (GABA,Glx)
	(1)	(2)	(3)	(4)	(5)
Intercept	−1.0* (−1.8, −0.2)	−1.1** (−1.7, −0.4)	−1.1** (−1.7, −0.5)	−2.0** (−3.2, −0.9)	−1.3* (−2.4, −0.2)
Trial	−0.001 (−0.02, 0.02)	−0.001 (−0.03, 0.03)	−0.001 (−0.03, 0.03)	−0.01 (−0.04, 0.03)	−0.01 (−0.05, 0.02)
GM			0.004 (−0.02, 0.03)		0.01 (−0.03, 0.05)
WM		0.2** (0.1, 0.4)	0.2** (0.1, 0.4)	−0.2 (−0.5, 0.04)	−0.3** (−0.6, −0.1)
a-tDCS	−0.5 (−1.3, 0.2)	0.04 (−0.6, 0.7)	0.04 (−0.6, 0.7)	0.4 (−0.5, 1.2)	0.3 (−0.6, 1.1)
E:I		−0.9** (−1.5, −0.3)		−1.3* (−2.4, −0.2)	
GABA			2.8** (1.0, 4.6)		4.9** (1.9, 7.8)
Glx			−0.3 (−1.0, 0.5)		0.4 (−1.2, 1.9)
Trial:a-tDCS	−0.01 (−0.02, 0.005)	−0.01 (−0.02, 0.01)	−0.01 (−0.02, 0.01)	−0.002 (−0.02, 0.01)	−0.003 (−0.02, 0.01)
Trial:EI		0.1 (−0.05, 0.2)	0.1 (−0.1, 0.2)	−0.3* (−0.5, −0.01)	−0.1 (−0.4, 0.2)
Trial:GABA			−0.04 (−0.1, 0.04)		−0.004 (−0.1, 0.1)
Trial:Glx		0.02 (−0.01, 0.05)		0.005 (−0.03, 0.04)	
a-tDCS:EI		0.8* (0.1, 1.4)		0.4 (−0.4, 1.1)	
a-tDCS:GABA			−1.5 (−3.3, 0.2)		−0.6 (−3.2, 1.9)
a-tDCS:Glx			0.7* (0.2, 1.3)		0.5 (−0.4, 1.5)
Trial:a-tDCS:EI		0.01* (0.002, 0.02)		0.005 (−0.01, 0.02)	
Trial:a-tDCS:GABA			−0.03 (−0.1, 0.01)		−0.000 (−0.05, 0.05)
Trial:a-tDCS:Glx			0.004 (−0.01, 0.01)		0.01 (−0.01, 0.03)
Observations	2,250	1,440	1,440	1,080	1,080
Bayesian Inf. Crit.	8,378.3	5,400.6	5,425.6	4,148.1	4,170.5
Note:					*p<0.05; **p<0.01

Table S9: **Experiment 2: Effect of M1 a-tDCS on long-term memory and interaction with M1/V1 neurochemistry.** All linear mixed-effect models use the normalised pointing error at 24-hours post-PA as the dependent variable. Model (1) assesses the effect of left M1 a-tDCS on the after-effect at 10-min. Model (2) assesses the interaction of left M1 E:I (Glx:GABA) with this effect. Model (3) decomposes the individual interaction of M1 GABA and Glx with the behavioural effect of a-tDCS effect on long-term retention. Models (4) and (5) are similar to models (2) and (3) but use V1 neurochemistry instead. The most important finding here is that M1 E:I significantly interacted with the effect of a-tDCS on long-term retention (a-tDCS:EI in Model 2), which could be decomposed into opposite interactions with GABA and Glx (a-tDCS:GABA and a-tDCS:Glx in Model 3). The same effect was not observed for V1 neurochemistry (Models 4 and 5). All models including MRS data also controlled for the fraction of grey and white matter within the voxel. *p<0.05; **p<0.01 (all two-tailed).
